# Coupling shRNA screens with single-cell RNA-seq identifies a dual role for mTOR in reprogramming-induced senescence

**DOI:** 10.1101/gad.297796.117

**Published:** 2017-10-15

**Authors:** Marieke Aarts, Athena Georgilis, Meryam Beniazza, Patrizia Beolchi, Ana Banito, Thomas Carroll, Marizela Kulisic, Daniel F. Kaemena, Gopuraja Dharmalingam, Nadine Martin, Wolf Reik, Johannes Zuber, Keisuke Kaji, Tamir Chandra, Jesús Gil

**Affiliations:** 1 Medical Research Council (MRC) London Institute of Medical Sciences (LMS), London W12 0NN, United Kingdom;; 2 Institute of Clinical Sciences (ICS), Faculty of Medicine, Imperial College London, London W12 0NN, United Kingdom;; 3 MRC Centre for Regenerative Medicine, University of Edinburgh, Edinburgh EH16 4UU, United Kingdom;; 4 WaferGen Biosystems, L-1526 Luxembourg, Luxembourg;; 5 Epigenetics Programme, The Babraham Institute, Cambridge CB22 3AT, United Kingdom;; 6 Research Institute of Molecular Pathology (IMP), Vienna Biocenter (VBC), Vienna 1030, Austria;; 7 MRC Human Genetics Unit, University of Edinburgh, Edinburgh EH4 2XU, United Kingdom

**Keywords:** SASP, senescence, iPSCs, single-cell RNA-seq, shRNA screens

## Abstract

Expression of the transcription factors OCT4, SOX2, KLF4, and cMYC (OSKM) reprograms somatic cells into induced pluripotent stem cells (iPSCs). Reprogramming is a slow and inefficient process, suggesting the presence of safeguarding mechanisms that counteract cell fate conversion. One such mechanism is senescence. To identify modulators of reprogramming-induced senescence, we performed a genome-wide shRNA screen in primary human fibroblasts expressing OSKM. In the screen, we identified novel mediators of OSKM-induced senescence and validated previously implicated genes such as *CDKN1A*. We developed an innovative approach that integrates single-cell RNA sequencing (scRNA-seq) with the shRNA screen to investigate the mechanism of action of the identified candidates. Our data unveiled regulation of senescence as a novel way by which mechanistic target of rapamycin (mTOR) influences reprogramming. On one hand, mTOR inhibition blunts the induction of cyclin-dependent kinase (CDK) inhibitors (CDKIs), including p16^INK4a^, p21^CIP1^, and p15^INK4b^, preventing OSKM-induced senescence. On the other hand, inhibition of mTOR blunts the senescence-associated secretory phenotype (SASP), which itself favors reprogramming. These contrasting actions contribute to explain the complex effect that mTOR has on reprogramming. Overall, our study highlights the advantage of combining functional screens with scRNA-seq to accelerate the discovery of pathways controlling complex phenotypes.

## Abstract (precis)

Aarts et al. developed an innovative approach that integrates single-cell RNA sequencing with a shRNA screen in primary human fibroblasts expressing OCT4, SOX2, KLF4, and cMYC to investigate the mechanism of action of the identified candidates. This approach unveiled regulation of senescence as a novel way by which mechanistic target of rapamycin (mTOR) influences reprogramming.

During normal development, cells gradually acquire more differentiated fates. Several strategies, referred to as cellular reprogramming, can reverse the natural direction of the differentiation process. The reprogramming of somatic cells to a pluripotent state can be achieved by either nuclear transfer (Gurdon 1962; Wilmut et al. 1997) or cellular fusion between somatic and embryonic stem (ES) cells (Tada et al. 2001). In addition, the seminal work of Yamanaka (Takahashi and Yamanaka 2006) showed that the expression of pluripotency-associated factors (OCT4, SOX2, KLF4, and cMYC, collectively referred to as OSKM) also could be used to reprogram somatic cells to an ES-like cell type known as induced pluripotent stem cells (iPSCs). This discovery holds great promise for practical applications in regenerative medicine. Gaining a greater understanding of cellular reprogramming will also provide clues about the mechanisms behind differentiation and dedifferentiation pertinent to normal development or cancer (Shi et al. 2017).

Reprogramming somatic cells to iPSCs is a long process with an overall low success rate. The inefficiency of reprogramming has suggested the existence of several barriers impeding iPSC generation. The mechanisms underlying barriers to cellular reprogramming have been investigated previously using functional screens (Qin et al. 2014; Sakurai et al. 2014; Yang et al. 2014; Cheloufi et al. 2015). An inherent technical difficulty of working with primary cells is that once in culture, they eventually undergo replicative or stress-induced senescence (Kuilman et al. 2010). To overcome this issue, genetic tricks to blunt senescence (for example, overexpressing hTERT or knocking down p53 expression) have sometimes been used to screen for genes limiting reprogramming (Qin et al. 2014), indirectly highlighting that senescence constitutes an intrinsic cellular barrier to iPSC generation (Krizhanovsky and Lowe 2009; Banito and Gil 2010). Key tumor suppressors such as p53, p16^INK4a^, or p21^CIP1^ control the senescence response to OSKM, and their inhibition increases reprogramming (Banito et al. 2009; Hong et al. 2009; Kawamura et al. 2009; Li et al. 2009; Marion et al. 2009; Utikal et al. 2009). However, little else is understood about the mechanisms governing senescence induction during reprogramming and how similar it is to other types of senescence.

Senescence is a cellular program that restrains the replication of damaged or old cells by imposing a stable cell cycle arrest. As part of the senescence program, cells undergo additional phenotypic alterations, including remodeling of their chromatin or secreting a complex mixture of factors known as the senescence-associated secretory phenotype (SASP) (Kuilman et al. 2010; Salama et al. 2014). Senescent cells are present in injured, preneoplastic, old, and diseased tissues and influence many phenotypes through the SASP (Coppe et al. 2010). In general, the accumulation of senescent cells is detrimental (Munoz-Espin and Serrano 2014), and their elimination ameliorates many age-related pathologies (Baker et al. 2016), whereas senescence induction limits fibrosis (Krizhanovsky et al. 2008) and cancer (Collado and Serrano 2010). Senescent cells found in old or injured tissues also create a permissive environment for in vivo reprogramming (Mosteiro et al. 2016). This has been linked to increased production of IL-6 as part of the SASP, as IL-6 facilitates reprogramming by activating cMYC and PIM1 (Brady et al. 2013). Given the physiological relevance of senescence and that it constitutes an intrinsic barrier for reprogramming, understanding how senescence is regulated during reprogramming is important. Genetic screening is a powerful tool that has been exploited with success to identify genes regulating senescence (Jacobs et al. 2000; Rowland et al. 2005; Acosta et al. 2008; Tordella et al. 2016; Wang et al. 2016) or cellular reprogramming (Qin et al. 2014; Sakurai et al. 2014; Yang et al. 2014).

In this study, we screened for shRNAs blunting reprogramming-induced senescence. An inherent limitation of pooled screens is the protracted process of retesting, validation, and characterization of identified candidates. To speed this up, we combined single-cell RNA sequencing (scRNA-seq) with shRNA screens. Using this approach, we discovered that, by regulating senescence, mechanistic target of rapamycin (mTOR) controls cell-intrinsic and cell-extrinsic mechanisms with opposing effects on reprogramming. Moreover, our study highlights the advantages of combining functional screens with scRNA-seq analysis.

## Results

### Exploring the senescence program induced by OSKM

**Figure 1.**
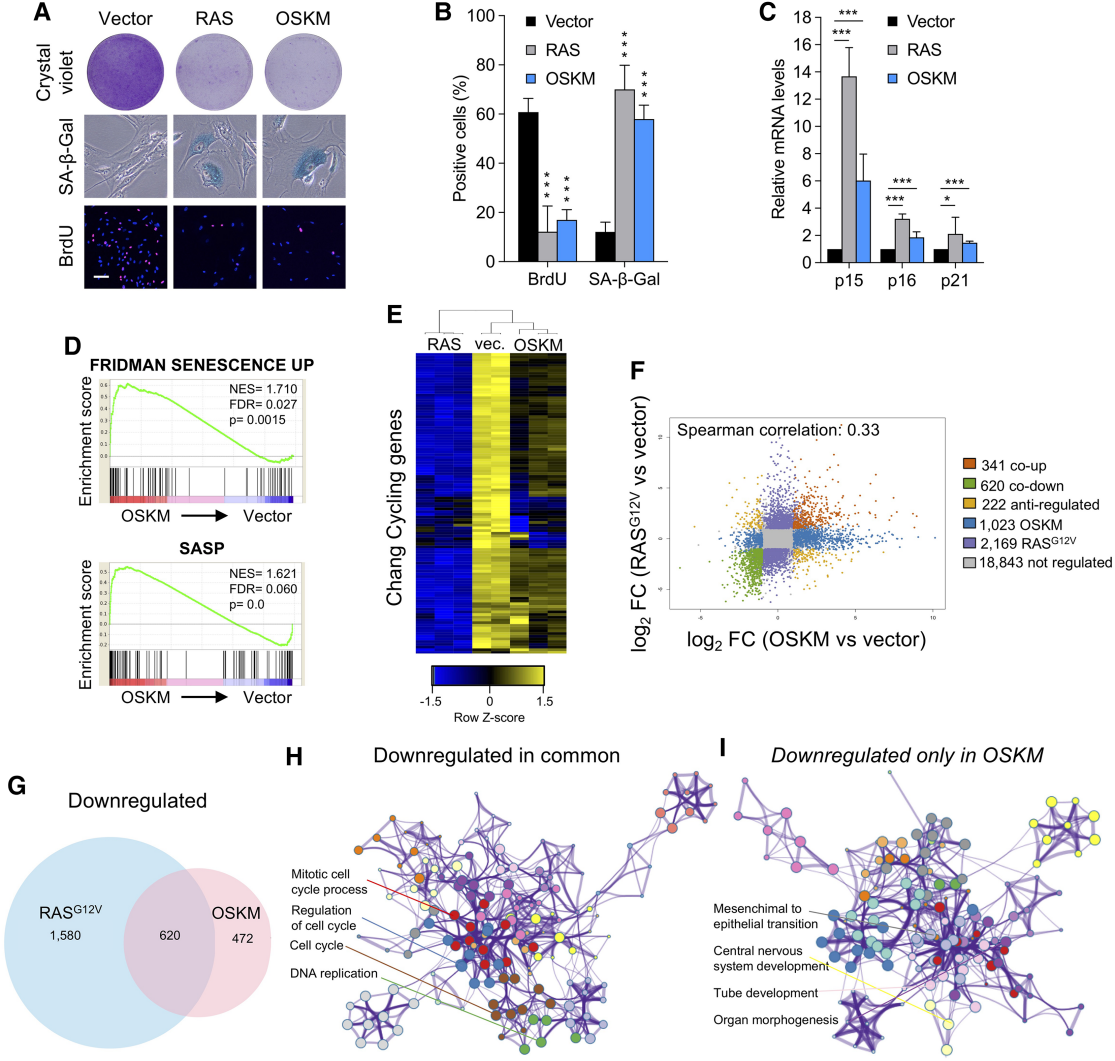
Expression of OSKM results in the induction of a characteristic senescence program in IMR90 human fibroblasts. (*A*) Senescence markers in IMR90 fibroblasts infected with control (vector), a polycistronic vector expressing OSKM or RAS. At 12 d after infection, senescence was assayed by crystal violet staining (*top*), senescence-associated β-galactosidase (SA-β-Gal) activity (*middle*), and BrdU incorporation after an 18-h pulse (*bottom*). Bar, 100 µM. (*B*) Quantification of BrdU and SA-β-Gal-positive cells after infection with the indicated vectors. (***) *P* < 0.001. (*C*) Quantitative RT–PCR (qRT–PCR) showing the mRNA expression levels of *CDKN2B* (encoding p15^INK4b^), *CDKN2A* (p16^INK4a^), and *CDKN1A* (p21^CIP1^) after infection with the indicated vectors. (*) *P* < 0.05; (***) *P* < 0.001. (*D*) Gene set enrichment analysis (GSEA) showing enrichment of signatures associated with senescence and the SASP in OSKM versus vector-expressing IMR90 cells. (NES) Normalized enrichment score. (*E*) Heat map showing gene expression of cell cycle genes (Chang et al. 2004) for IMR90 cells infected with vector, RAS, and OSKM. Log_2_ expression values (rlog) were row-normalized using *Z*-scores, and only genes that have higher expression in RAS or OSKM compared with vector are shown in the heat map. Both genes and samples were clustered using hierarchical clustering. (*F*) Scatter plot showing log_2_ fold change (FC) in gene expression between RAS versus vector and OSKM versus vector. Genes changing (false discovery rate [FDR] < 0.05; log_2_ fold change < −1 or log_2_ fold change > 1) are shown in color. (*G*) Venn diagram showing common down-regulated genes between RAS versus vector and OSKM versus vector. Down-regulated genes were defined as those with log_2_ fold change < −1 and FDR < 0.05. (*H*,*I*) Gene ontology (GO) term analysis of common genes down-regulated upon OSKM- and RAS-induced senescence (*H*) or down-regulated only in OSKM-induced senescence (*I*). First, for each senescence type, genes differentially regulated compared with control (vector) by log_2_ fold change < −1 (*P* < 0.05) were selected. Next, common genes were uploaded to the online bioinformatics database Metascape (http://metascape.org) for GO term detection and clustering. Same-colored dots fall into a function category similar to the given title. Only statistically significant categories (*P* < 0.05) are shown.

Expression of the reprogramming factors (OSKM) in IMR90 human fibroblasts causes a senescence-like growth arrest (Fig. 1A,B) that constitutes an intrinsic barrier to reprogramming (Banito et al. 2009). Similar to oncogenic RAS^G12V^, the expression of OSKM induces the cyclin-dependent kinase (CDK) inhibitors (CDKIs) p15^INK4b^, p16^INK4a^, and p21^CIP1^, which are involved in implementing the stable growth arrest associated with senescence (Fig. 1C).

To better characterize OSKM-induced senescence, we took advantage of RNA sequencing (RNA-seq). Gene set enrichment analysis (GSEA) found signatures for senescence and the SASP significantly enriched in the transcriptome of cells expressing OSKM (Fig. 1D). Other signatures showed a similar association with OSKM- and RAS-induced senescence. For example, TGF-β-dependent signatures were up-regulated in both types of senescence (Supplemental Fig. S1A). In some instances, although the effect of RAS or OSKM expression was qualitatively equivalent, the strength of the responses differed. For example, although signatures associated with proliferation were down-regulated upon RAS or OSKM expression (Supplemental Fig. S1B), a stronger growth arrest was associated with RAS expression (Fig. 1E). Overall, we observed a moderate correlation between the transcriptional changes induced by RAS and OSKM (Spearman correlation = 0.33) (Fig. 1F). Among the genes regulated in common (Fig. 1G; Supplemental Fig. S1C), gene ontology (GO) analysis highlighted several senescence processes (such as down-regulation of terms related to mitosis and cell cycle or up-regulation of inflammatory responses) (Fig. 1H; Supplemental Fig. S1D). Besides these commonalities, the specific nature of the OSKM and RAS transcriptional programs was also evident. For example, GO terms associated with epithelial-to-mesenchymal transition and development and differentiation processes were preferentially regulated by OSKM rather than RAS expression (Fig. 1I; Supplemental Fig. S1E). Overall, the above results confirm that OSKM expression induces a senescence program with distinctive characteristics.

### A screen for shRNAs regulating OSKM-induced senescence

**Figure 2.**
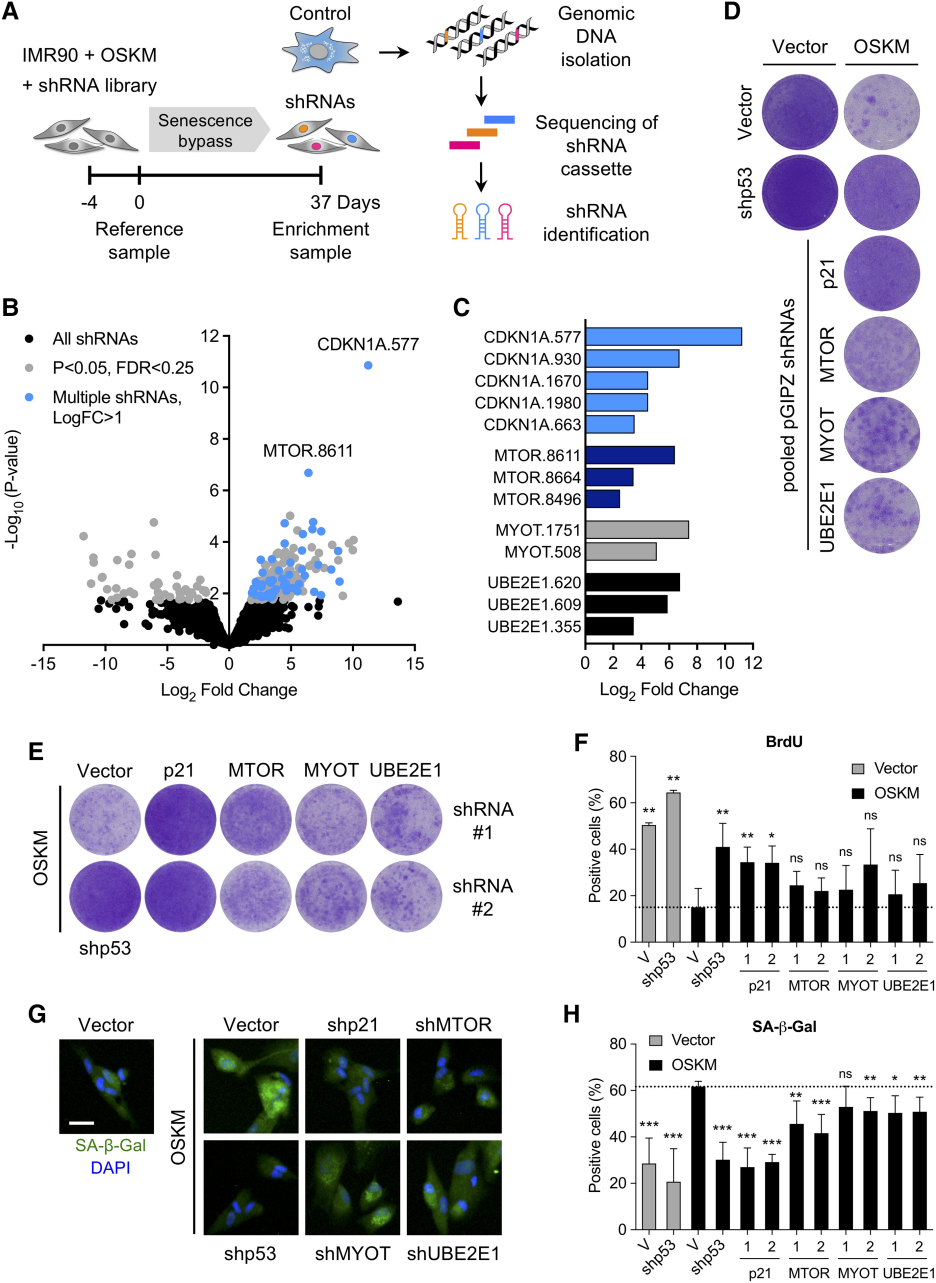
An shRNA screen identifies modulators of reprogramming-induced senescence. (A) Time line and strategy of a secondary shRNA enrichment screen. IMR90 fibroblasts were infected with an OSKM expression vector followed by a pooled shRNA library in duplicate. Samples for analysis of shRNA library representation were taken at regular intervals over a 37-d culture period. Two independent biological shRNA screens were performed. (*B*) Volcano plot depicting the changes in representation (log_2_ fold change; *X*-axis) and significance (−log_10_-converted *P*-value; *Y*-axis) of each shRNA in the library at day 37 versus day 0. Total library (black; 3153 shRNAs), enriched shRNAs (gray; *P* < 0.05; FDR < 0.25; 229 shRNAs), and candidates with multiple shRNAs (blue; log_2_ fold change > 1; 52 shRNAs) are shown. The top shRNAs targeting CDKN1A and MTOR are highlighted. EdgeR statistical analysis was used to combine and batch-correct data from two independent biological screens. (*C*) Significantly enriched (log_2_ fold change) shRNAs for CDKN1A, MTOR, MYOT, and UBE2E1. (*D*) Initial validation of candidates identified in the secondary screen. IMR90 fibroblasts were infected with control or OSKM expression vector and pooled pGIPZ shRNAs against the indicated candidates. Cell proliferation was assayed by crystal violet staining. (*E*) IMR90 fibroblasts were infected with OSKM followed by two different shRNAs against p21, MTOR, MYOT, and UBE2E1. At 12 d after infection, cells were seeded at low density. After 16 d, plates were stained with crystal violet. Images are from a representative experiment. (*F*) IMR90 fibroblasts were infected with empty vector (gray bars) or OSKM (black bars) followed by shp53 or two different shRNAs against p21, MTOR, MYOT, and UBE2E1 or control vector (V). At 12 d after infection, cells were seeded in 96-well plates and cultured for another 5 d. The percentage of BrdU-positive cells was determined by immunofluorescence after an 18-h pulse with BrdU. Error bars represent the SD of three independent experiments. (*) *P* < 0.05; (**) *P* < 0.01; (ns) not significant. (*G*) IMR90 fibroblasts were treated as described in *F*. At 12 d after infection, cells were seeded in 96-well plates. The next day, SA-β-Gal activity was determined by fluorescence staining. Representative images are shown for cells infected with the indicated vectors. Bar, 30 µm. (*H*) Quantification of SA-β-Gal-positive cells treated as described in *G*. (Gray bars) Cells infected with empty vector control (V); (black bars) cells infected with OSKM vector. Error bars represent the SD of at least three independent experiments. (*) *P* < 0.05; (**) *P* < 0.01; (***) *P* < 0.001; (ns) not significant.

To identify genes that regulate OSKM-induced senescence, we screened a shRNA library comprised of ∼58,000 shRNAs (Supplemental Fig. S2A). IMR90 fibroblasts were transduced with a retroviral vector expressing OSKM followed by lentiviral transduction with the shRNA library. Cells were passaged to enrich for shRNAs blunting the senescence growth arrest. In parallel, cells were infected with a shRNA against p53 (shp53), which prevents the senescence growth arrest (Supplemental Fig. S2B). Integrated shRNAs were identified, and their enrichment was assessed using next-generation sequencing (NGS) (Supplemental Fig. S2C). Five-hundred-fifty-four candidate genes were selected using the criteria described in Supplemental Figure S2A. A shRNA library targeting these candidates (average coverage of six shRNAs per gene; 3153 shRNAs in total) was generated and screened similarly (Fig. 2A). Statistical analysis identified shRNAs significantly enriched with time in OSKM-expressing cells (day 37 vs. day 0) (Fig. 2B,C). After retesting shRNAs targeting the top screen candidates, we found that infection with shRNAs targeting four of these genes (*CDKN1A*, *MTOR*, *MYOT*, and *UBE2E1*) resulted in a consistent bypass of OSKM-induced senescence (Fig. 2D; Supplemental Fig. S2D).

To validate the screen results, IMR90 fibroblasts were infected with OSKM and two individual shRNAs targeting each candidate. We assessed the ability of the different shRNAs to knock down their targets (Supplemental Fig. S3A–C). *MYOT* expression was below the detection limit, and its knockdown could not be confirmed despite independent shRNAs reproducing the bypass of senescence phenotype (data not shown). The ability of shRNAs targeting *CDKN1A*, *MYOT*, *MTOR*, and *UBE2E1* to prevent OSKM-induced senescence was confirmed by increased proliferation (Fig. 2E), a higher percentage of cells incorporating BrdU (Fig. 2F; Supplemental Fig. S3D), and a decrease in the percentage of senescence-associated β-galactosidase (SA-β-Gal)-positive cells when compared with IMR90 cells infected with OSKM and a control vector (Fig. 2G,H; Supplemental Fig. S3E). Since p21^CIP1^ has been implicated previously in controlling reprogramming-induced senescence (Banito et al. 2009), these results suggest that our screen successfully identified genes regulating senescence.

### scRNA-seq as an approach to facilitate the analysis of shRNA screens

An important bottleneck in genetic screens such as the one described in this study is the retesting, validation, and characterization of the identified candidates. The advent of scRNA-seq has made it possible to analyze gene expression at a cellular level rather than relying on average levels from cell populations (Tanay and Regev 2017). We hypothesized that by performing scRNA-seq in parallel with measuring shRNA enrichment in bulk populations, the characterization of our screen candidates could be accelerated.

**Figure 3.**
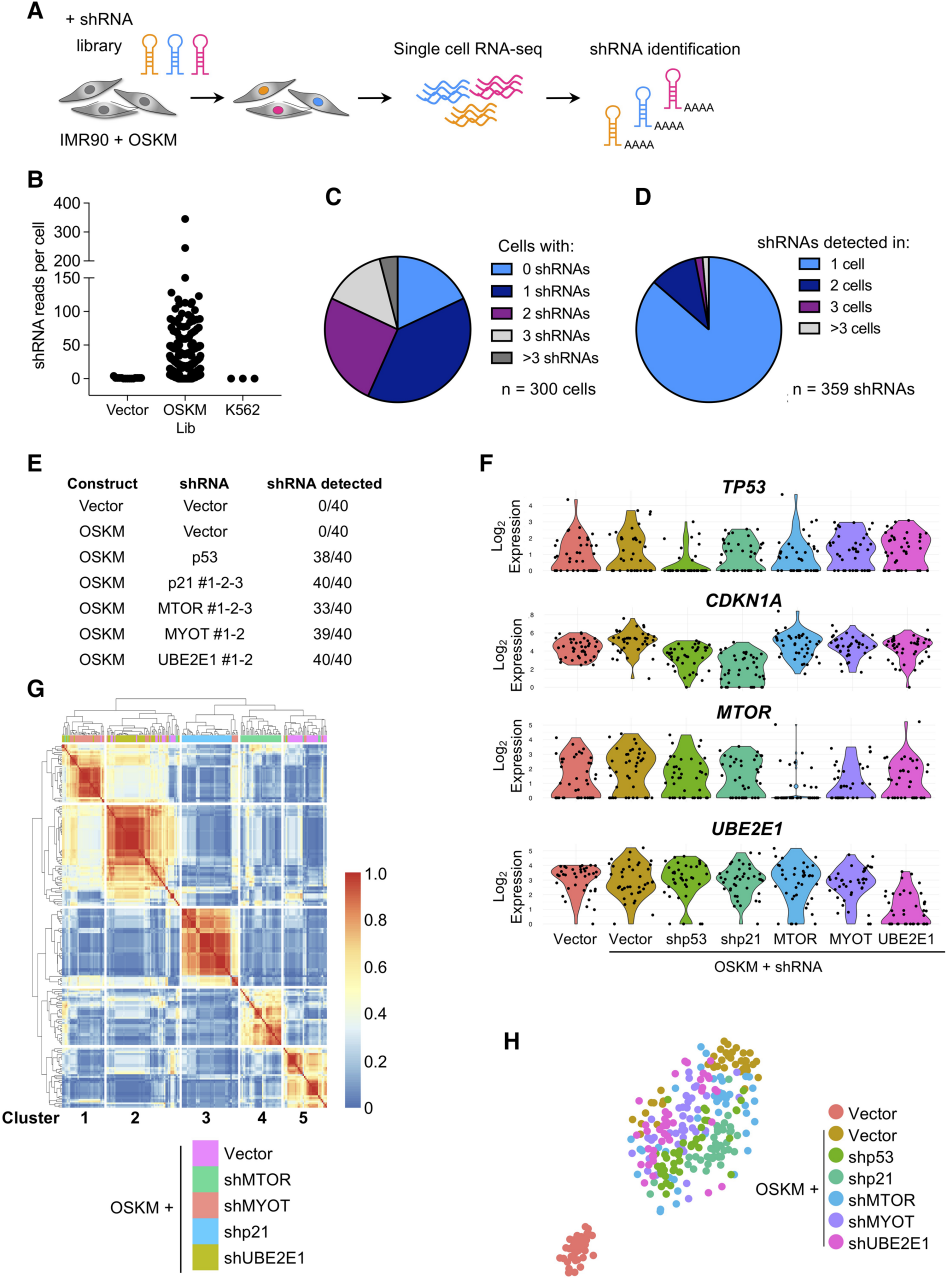
Coupling scRNA-seq with shRNA assignment identifies expression profiles associated with gene knockdown. (*A*) Strategy of scRNA-seq analysis of IMR90 cells infected with OSKM and an shRNA library. RNA-seq libraries were prepared using the ICELL8 single-cell system. (*B*) The number of shRNA-specific reads plotted for IMR90 cells infected with empty shRNA vector (no shRNA-specific insert; *n* = 50), OSKM and shRNA library cells (OSKM/Lib; *n* = 300), and K562 control cells that were not exposed to shRNA vectors (*n* = 50). (*C*) Pie chart showing the number of different shRNAs that could be detected per cell. One or more shRNAs could be detected in 82% of the single-cell libraries (246 out of 300), while 18% (54 out of 300) had no detectable shRNA reads. (*D*) Pie chart showing the occurrence of the 359 different shRNAs that were detected in 300 OSKM/Lib RNA-seq libraries. Overall, 86% (310 out of 359) of the shRNAs were found uniquely in one cell, while 14% (49 out of 359) of the hairpins were detected in two or more cells. (*E*) Experimental setup of the scRNA-seq experiment. Vector or OSKM-expressing IMR90 cells were infected with the indicated shRNAs. For each condition, 40 single cells were used for scRNA-seq analysis. RNA-seq libraries were prepared using the ICELL8 single-cell system (WaferGen Biosystems). (*F*) Violin plots of *TP53*, *CDKN1A*, *MTOR*, and *UBE2E1* mRNA expression of single cells infected with the indicated constructs. (*G*) Heat map and clustering analysis of differentially expressed genes of OSKM-expressing cells infected with vector, shp21, shMTOR, shMYOT, and shUBE2E1 resulted in five clusters, each enriched for cells containing shRNAs targeting a different gene. (*H*) The *t*-distributed stochastic neighbor embedding (*t*-SNE) plot of the 280 single cells depicts the separation into shp53, shMTOR, shCDKN1A, shMYOT, and shUBE2E1 relative to vector and OSKM control cells.

To evaluate this approach, we first assessed the accuracy of detecting shRNAs in single cells (Fig. 3A). The shRNAs were embedded in miR-E, an improved miR-30-based backbone (Fellmann et al. 2013), which is expressed from a RNA polymerase II (Pol II)-dependent promoter (Dickins et al. 2005). While most shRNA transcripts use the polyadenylation (poly-A) signal in the 3′ long terminal repeat (LTR) of the virus, we noted the presence of two putative poly-A signals (ATTAAA) in the 3′ context of miR-E, raising the possibility that protocols relying on poly-A priming could detect those transcripts. To call the shRNAs, we used reads that mapped to the shRNA-specific sequence and overlapped with the miR-E backbone (Supplemental Fig. S4A). The shRNA reads correlated with the total number of reads per cell (Supplemental Fig. S4B) and were detected only in cells transduced with shRNAs (Fig. 3B). Most cells contained a single shRNA (116 out of 300 cells) (Fig. 3C), and most shRNAs were present in just one cell (310 out of 359 shRNAs) (Fig. 3D).

Next, we performed scRNA-seq of IMR90 cells infected with OSKM and specific shRNAs (as summarized in Fig. 3E). Cells were infected independently with different shRNAs targeting each screen candidate (three shRNAs targeting *MTOR* and *CDKN1A* and two targeting *MYOT* and *UBE2E1*, respectively). The scRNA-seq information allowed us to assign shRNAs to most of the cells (Fig. 3E), and transcriptome analysis confirmed the down-regulation of *TP53*, *CDKN1A*, *MTOR*, and *UBE2E1* in cells infected with the corresponding shRNAs (Fig. 3F). Moreover, *CDKN1A*, a p53 target gene, was also down-regulated in OSKM–shp53 cells. We could not detect *MYOT* expression, as in our previous experiments.

Finally, to understand whether the scRNA-seq data could identify expression differences associated with the knockdown of the different candidates, we performed unsupervised hierarchical clustering (Fig. 3G; Supplemental Fig. S4C). The cells segregated into five stable clusters, each enriched for cells infected with shRNAs targeting a different gene. We used *t*-distributed stochastic neighbor embedding (*t*-SNE) analysis to further characterize the different subclusters and observed that the biggest determinant was the difference between OSKM-induced senescent versus growing (vector) cells (Fig. 3H). Nonetheless, differences associated with the knockdown of the screen candidates were detected, as exemplified by the projection of selected marker genes identified by clustering (Supplemental Fig. S4D).

### Coupling scRNA-seq to the shRNA screen identifies profiles associated with *MTOR* knockdown

**Figure 4.**
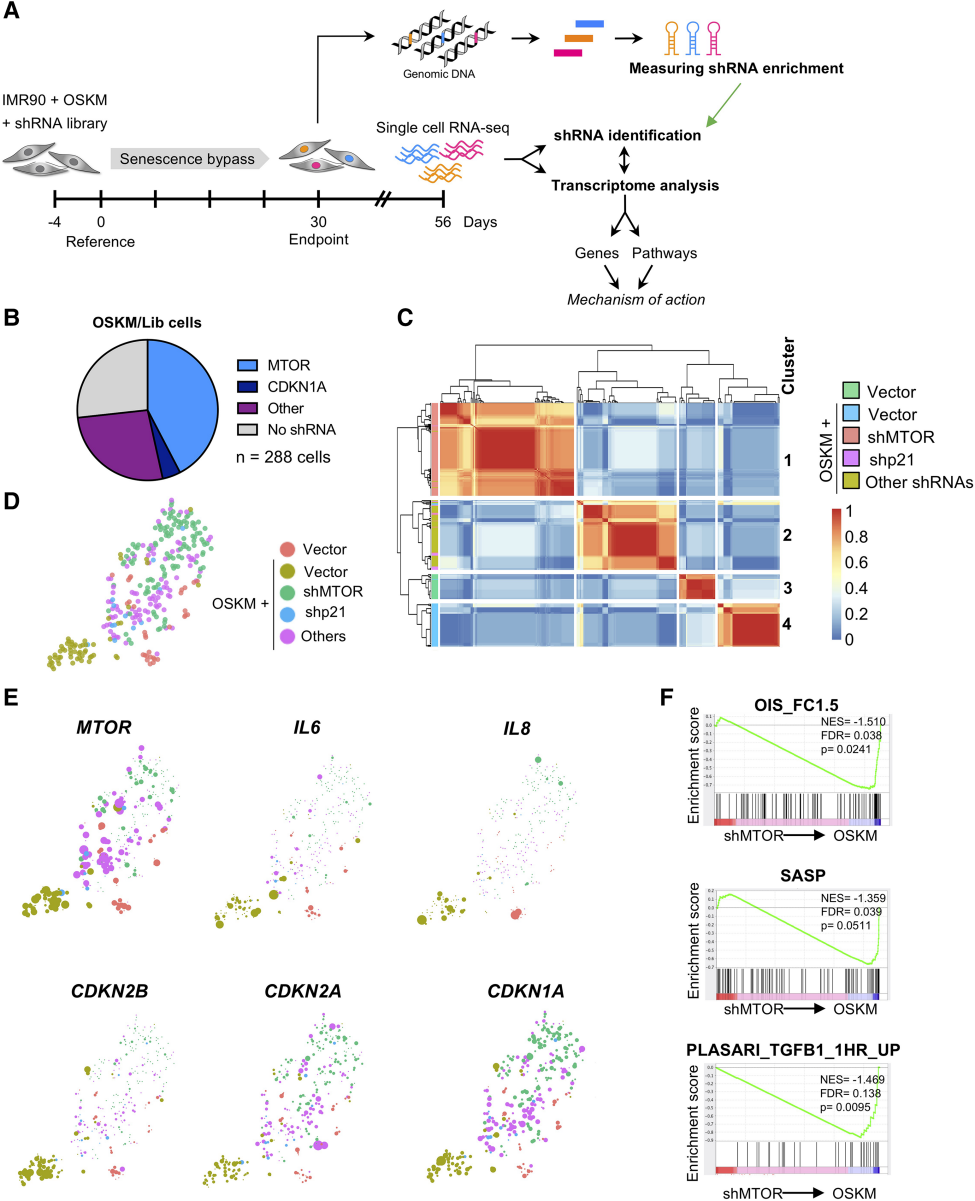
Integrating scRNA-seq analysis with an shRNA screen. (*A*) Time line and strategy of a secondary shRNA enrichment screen combined with scRNA-seq. IMR90 fibroblasts were infected as described in Figure 2A. From the second repeat screen, 288 single cells were sorted from one replicate at day 56 for transcriptome analysis by RNA-seq in parallel with genomic DNA isolation for shRNA enrichment analysis. scRNA-seq libraries were prepared using the Smart-seq2 protocol (Picelli et al. 2014). (*B*) Pie chart representing the shRNA occurrence among 288 single-cell libraries. The shRNA read count threshold was set at ≥10 for identification of shRNAs. (*C*) Heat map and clustering analysis of differentially expressed genes of OSKM/Lib cells compared with vector and OSKM cells results in separation of OSKM-expressing cells containing an shRNA against *MTOR* versus other shRNAs. (*D*) The *t*-SNE plot of the 288 single OSKM/Lib cells depicts the separation into shMTOR, shCDKN1A, and other shRNAs relative to vector and OSKM control cells. (*E*) Projection of *MTOR*, *IL6*, *IL8*, *CDKN1A*, *CDKN2A*, and *CDKN2B* onto the *t*-SNE plot from *D* is shown. (*F*) GSEA showing loss of signatures associated with senescence, the SASP, and TGF-β in OSKM–shMTOR cells versus OSKM control cells. (NES) Normalized enrichment score.

Taking advantage of the approach described above, we sorted 288 cells from the screen to carry out scRNA-seq. In parallel, we isolated genomic DNA for identification of shRNAs by NGS (Fig. 4A). At an arbitrary cutoff of >10 shRNA reads, we identified shRNAs in 211 out of 288 cells. Among those, we identified 122 out of 211 cells with shRNAs targeting *MTOR* and 12 out of 211 cells with shRNAs targeting *CDKN1A*, two of the screen hits (Fig. 4B). As expected, *MTOR* expression was significantly down-regulated in cells expressing the MTOR shRNA (Supplemental Fig. S5A). Next, we clustered the scRNA-seq data derived from the screen (Fig. 4C). The transcriptomes stably segregated into four clusters that were comprised mainly of vector cells, OSKM cells, OSKM–shMTOR cells, and cells with shRNAs that were not identified as enriched in the screen (other shRNAs) (Fig. 4C; Supplemental Fig. S5B). Using *t*-SNE, we visualized cell separation (Fig. 4D) and the expression of genes differentially expressed in distinct cell populations (Supplemental Fig. S5C).

The scRNA-seq data helped us understand how mTOR regulates OSKM-induced senescence and place it in the context of reprogramming. Cells with reduced *MTOR* expression showed reduced levels of different CDKIs (such as *CDKN2A*, *CDKN1A*, and, most notably, *CDKN2B*) (Fig. 4E). In addition, cells bearing shRNAs targeting *MTOR* down-regulated multiple SASP components, including *IL6*, *IL8*, and *INHBA* (Fig. 4E; data not shown). Moreover, GSEA showed that signatures related to senescence, SASP, and TGF-β, among others, were significantly down-regulated in OSKM–shMTOR cells (Fig. 4F; Supplemental Fig. S5D). Therefore, scRNA-seq served to identify candidate genes and pathways that could explain how MTOR regulates OSKM-induced senescence.

### TGF-β-mediated p21^CIP1^ induction contributes to OSKM-induced senescence

**Figure 5.**
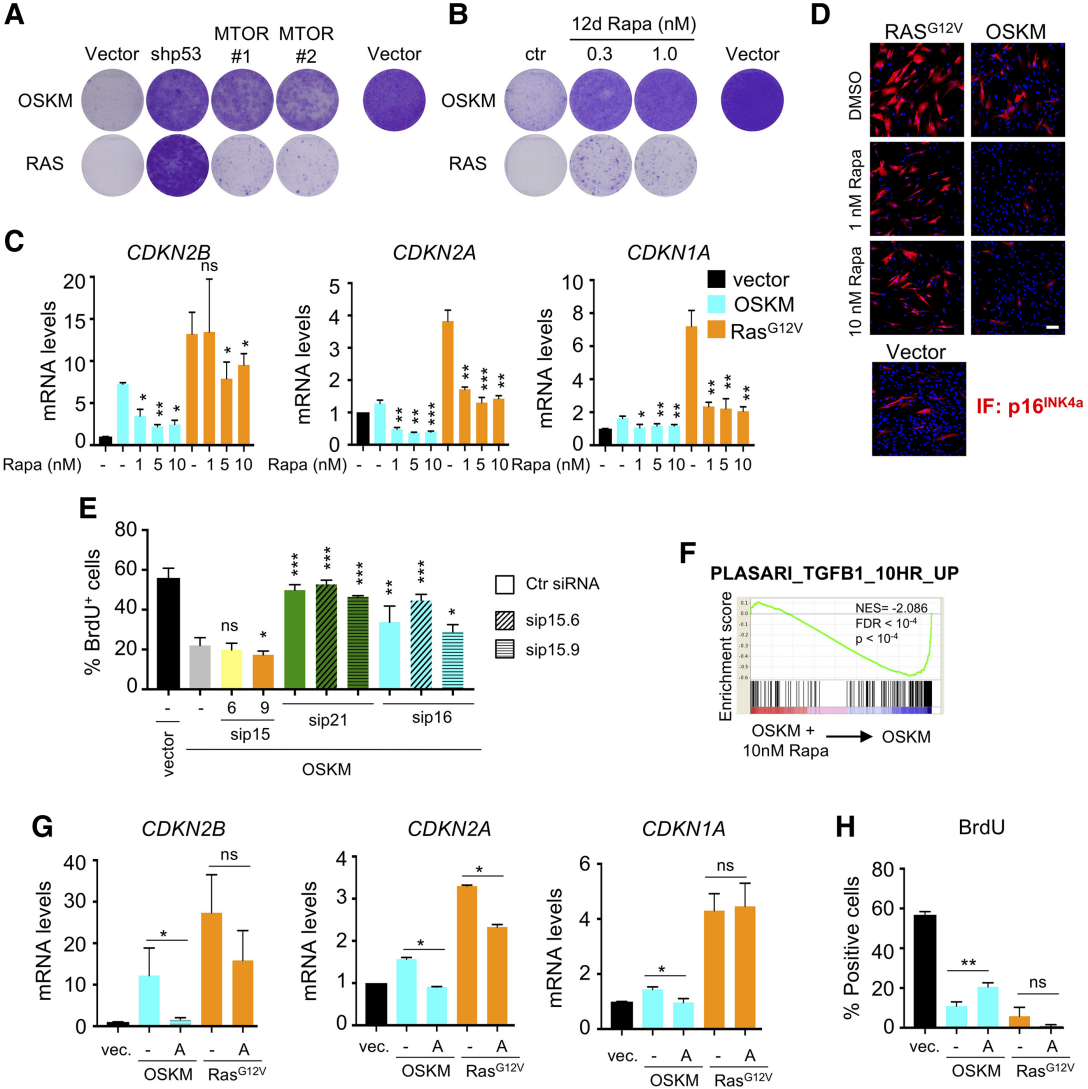
Investigating how mTOR inhibition affects OSKM-induced senescence. (*A*) Knockdown of *MTOR* by two different shRNAs prevented the growth arrest induced by OSKM (*top* row), but not by RAS (*bottom* row), as measured by crystal violet staining. (*B*) Inhibition of mTOR by rapamycin prevents the growth arrest induced by OSKM (*top* row) but not by RAS (*bottom* row). IMR90 fibroblasts were infected with OSKM or RAS and treated with 0.3 and 1.0 nM rapamycin (Rapa) the next day. At 12 d after infection, cells were seeded at low density and cultured for 16 more days in the absence of rapamycin before plates were stained with crystal violet. (*C*,*D*) Inhibition of mTOR by rapamycin blunts the induction of CDKIs, but the levels revert back to basal only in OSKM-induced senescence. IMR90 fibroblasts were infected with empty vector or OSKM- or RAS-expressing vectors and were treated the next day with DMSO (−) or increasing doses of rapamycin. At day 10 after infection, the cells were collected for qRT–PCR analysis of mRNA expression (*C*) or immunofluorescence (*D*) of the indicated CDKIs. Error bars represent the SD of three independent experiments. (*) *P* < 0.05; (**) *P* < 0.01; (***) *P* < 0.001; (ns) not significant. Bar, 100 μm. (*E*) *CDKN2B*, *CDKN2A*, and *CDKN1A* are up-regulated by OSKM expression, but only the latter two are necessary for OSKM-induced growth arrest. IMR90 fibroblasts were infected with empty vector or OSKM-expressing vector and, 2 d later, transfected with scramble siRNA (−), the indicated siRNAs, or combinations of siRNAs. BrdU quantification was performed at day 7 after infection. Error bars represent the SD of three independent experiments. (*) *P* < 0.05; (**) *P* < 0.01; (***) *P* < 0.001; (ns) not significant. (*F*) GSEA showing loss of a TGF-β signature in OSKM cells treated with 10 nM rapamycin versus OSKM cells. (NES) Normalized enrichment score. (*G*) Signaling via TGF-β RI kinase is necessary for the induction of the three CDKIs in OSKM-induced senescence but not RAS-induced senescence. IMR90 fibroblasts were infected with empty vector or OSKM- or RAS-expressing vectors and treated the next day with DMSO (−) or 23 nM TGF-β RI kinase inhibitor II (A; Calbiochem, 616452). At day 10 after infection, the cells were collected for qRT–PCR analysis of mRNA expression of the indicated CDKIs. Error bars represent the SD of three independent experiments. (*) *P* < 0.05; (ns) not significant. (*H*) Signaling via TGF-β RI kinase is necessary for OSKM-induced growth arrest but not RAS-induced growth arrest. IMR90 fibroblasts were infected with empty vector or OSKM- or RAS-expressing vectors and treated with DMSO (−) or 23 nM TGF-β RI kinase inhibitor II (A; Calbiochem, 616452). BrdU quantification was performed at day 9 after infection. Error bars represent the SD of three independent experiments (**) *P* < 0.01; (ns) not significant.

To investigate how MTOR regulates senescence, we compared the effect of knocking down MTOR in OSKM- or RAS-induced senescence. While *MTOR* depletion prevented OSKM-induced senescence, it did not affect the RAS-induced senescent arrest (Fig. 5A). This was consistent with our previous data (Herranz et al. 2015). Indeed, treatment with the mTOR inhibitor rapamycin prevented the arrest caused by OSKM but not that induced by RAS expression (Fig. 5B). Transcriptome analysis confirmed that rapamycin restored expression of proliferation-associated genes in OSKM-expressing but not RAS-expressing cells (Supplemental Fig. S6A). Based on the scRNA-seq data, we examined the expression of *CDKN2A*, *CDKN2B*, and *CDKN1A* to understand how mTOR inhibition prevents OSKM-induced (but not RAS-induced) senescence. Consistent with the scRNA-seq data, *CDKN2B* was highly induced by OSKM, and this induction was diminished upon mTOR inhibition (Fig. 5C). A similar pattern was observed for *CDKN2A* and *CDKN1A*, although the induction was more modest. RAS expression induced the expression of all three CDKIs more robustly. Although treatment with rapamycin blunted the up-regulation of all three CDKIs in response to either OSKM or RAS expression, their expression only came back to (or below) basal levels in OSKM-expressing cells treated with rapamycin (Fig. 5C). Analysis of p16^INK4a^ expression by Western blot or immunofluorescence analysis confirmed both a lower induction of p16^INK4a^ levels and a stronger decrease upon rapamycin treatment in OSKM-expressing cells than in RAS-expressing cells (Fig. 5D; Supplemental Fig. S6B).

We next examined the relative contribution of CDKN1A, CDKN2A, and CDKN2B to OSKM-induced senescence. Despite the extent of *CDKN2B* induction (Figs. 4E, 5C), *CDKN2B* knockdown with two independent siRNAs did not prevent the growth arrest caused by OSKM (Fig. 5E; Supplemental Fig. S6C; data not shown). On the other hand, knocking down *CDKN1A* or *CDKN2A* blunted the OSKM-induced arrest, but again, no additional effect was observed upon *CDKN2B* knockdown (Fig. 5E; Supplemental Fig. S6D,E).

To further understand the mechanism behind the MTOR-dependent induction of senescence by OSKM, we searched for pathways that could explain the induction of CDKIs. In this regard, the scRNA-seq data and the follow-up analysis highlighted that TGF-β signaling was induced by OSKM (Supplemental Fig. S1A) and affected by shMTOR (Fig. 4F) or rapamycin treatment (Fig. 5F). Both *CDKN1A* and *CDKN2B* are well-known effectors of the TGF-β pathway (Reynisdottir et al. 1995), and p16^INK4a^ can be induced by TGF-β during senescence (Vijayachandra et al. 2009). Interestingly, TGFBRI inhibition prevented the up-regulation of *CDKN2B*, *CDKN1A*, and *CDKN2A* by OSKM expression but had just minimal impact in response to RAS expression (Fig. 5G). Moreover, inhibition of TGFBRI signaling blunted the growth arrest triggered by OSKM (Fig. 5H). The above results suggest that increased TGF-β signaling is important to induce p21^CIP1^ and other CDKIs in response to OSKM induction and that mTOR inhibition interferes with this pathway to prevent senescence.

### Regulation of senescence by mTOR has opposing cell-intrinsic and cell-extrinsic effects on reprogramming

**Figure 6.**
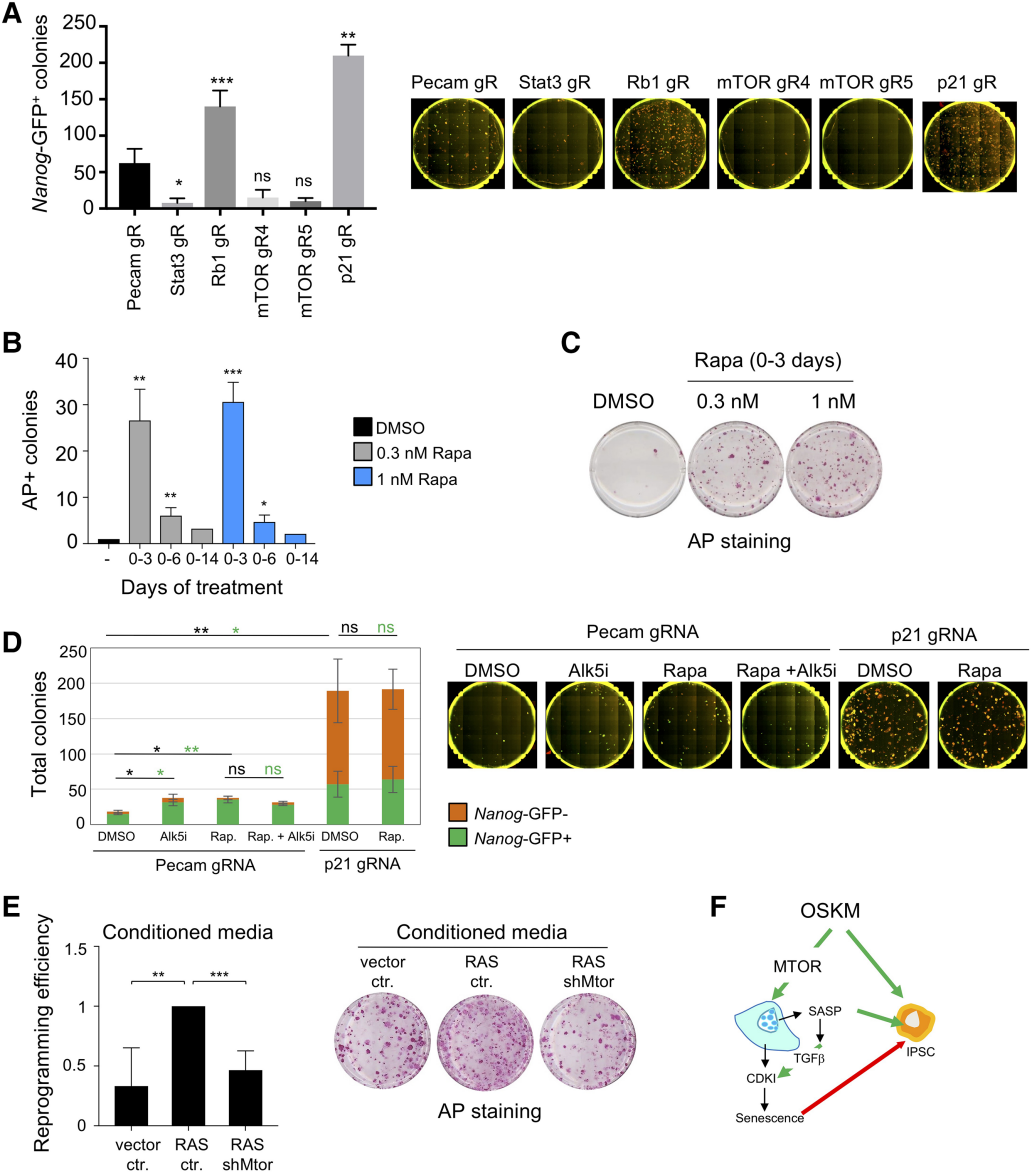
Dual effect of mTOR inhibition in iPSC reprogramming. (*A*) Reprogramming of Cas9-expressing TNG MKOS MEFs was initiated 1 d after transfection with a *piggyBac* transposon carrying an inducible MKOS cassette and the indicated gRNA expression gRNA expression cassette. Numbers of total and Nanog-GFP^+^ colonies were counted on day 14. See Supplemental Figure S7, B and C, for an expanded version of this figure. Error bars represent the SD of three independent experiments. (*) *P* < 0.05; (**) *P* < 0.01; (***) *P* < 0.001; (ns) not significant. (*B*) Dose- and time-dependent effect of rapamycin on reprogramming. Rapamycin (0.3 and 1.0 nM) was added for 3, 6, or 14 d after MKOS induction. Resulting iPSC colonies were stained for alkaline phosphatase (AP) after 14 d. Data were normalized to untreated cells. Error bars represent the SD of three independent experiments (days 0–3 and 0–6). Data from one experiment are shown for days 0–14. (*) *P* < 0.05; (**) *P* < 0.01; (***) *P* < 0.001. (*C*) Dose-dependent effect of rapamycin on reprogramming. Rapamycin (0.3 and 1.0 nM) was added for 3 d after MKOS induction. Resulting iPSC colonies were stained for alkaline phosphatase after 14 d. (*D*) Reprogramming of Cas9-expressing Nanog-GFP MEFs was performed after transfection of a *piggyBac* transposon carrying inducible MKOS-ires-mOrange and U6-driven gRNA expression cassettes in the presence or absence of 500 nM TGF-β RI inhibitor (Alk5i; Tocris, A83-01) and 5 nM rapamycin (Rap). Numbers of total and Nanog-GFP^+^ colonies were counted on day 14. Error bars represent the SD of three independent experiments. (*) *P* < 0.05; (**) *P* < 0.01; (ns) not significant. (Green) Statistics for Nanog^+^ colonies; (black) statistics for total number of colonies. (*E*) Reprogramming efficiency of transgenic MKOS MEFs treated with conditioned medium (CM) from MEFs infected with control vector, RAS, or RAS and shRNAs against *Mtor*. CM was collected after 3 d and reconstituted with 4× concentrated reprogramming medium before being added to the transgenic MEFs. Alkaline phosphatase-positive (AP^+^) colonies were counted, and data were normalized to cells treated with CM from RAS/ctr. Error bars represent the SD of four independent experiments. (**) *P* < 0.01; (***) *P* < 0.001. Representative images are shown. (*F*) Scheme summarizing the dual action of MTOR on regulation of senescence and reprogramming of iPSCs. (Green arrows) induction; (red arrow) inhibition.

Senescence imposes a cell-intrinsic barrier to reprogramming (Banito et al. 2009). To investigate how the identified regulators of OSKM-induced senescence affect iPSC generation, we performed reprogramming of mouse embryonic fibroblasts (MEFs) with constitutive Cas9 expression and a *Nanog*-GFP reporter using a piggyBac transposon carrying doxycycline (dox)-inducible MKOS-ires-mOrange and a U6-driven guide RNA (gRNA) expression cassette. The effectiveness of the gRNA sequences was confirmed by infecting Cas9-expressing MEFs with lentiviral gRNA expression vectors (Supplemental Fig. S7A; Tzelepis et al. 2016). As controls, we used gRNAs targeting Pecam (not affecting reprogramming), Stat3 (negative effect), and Rb1 (positive effect). In line with previous evidence, knocking out p21^CIP1^ increased the numbers of both total and Nanog-positive colonies (Fig. 6A; Supplemental Fig. S7B,C). Unexpectedly knocking *Myot* or *Ube2e1* out did not affect reprogramming (Supplemental Fig. S7B,C). On the contrary, *Mtor* knockout had a negative impact on reprogramming (Fig. 6A; Supplemental Fig. S7B,C). Although there may be many other possible explanations, these results suggest that preventing senescence per se does not necessarily result in an increase in reprogramming.

It had been shown previously that mTOR exerts a dose- and time-dependent effect on reprogramming (Chen et al. 2011), which might explain the detrimental effect observed upon its knockout. To further dissect the role of Mtor in reprogramming and understand the contribution of senescence regulation, we took advantage of the mTOR inhibitor rapamycin. Short treatment with nanomolar doses of rapamycin enhanced reprogramming, while more sustained treatment decreased reprogramming (Fig. 6B,C).

Regulation of reprogramming by mTOR has been connected to its ability to control autophagy (Wang et al. 2013; Wu et al. 2015), but, so far, no link has been established with the ability of mTOR to regulate senescence. Since we showed that mTOR regulates senescence via TGF-β-dependent regulation of p21^CIP1^ and other CDKIs, we examined how interfering with this pathway affected reprogramming. While a TGF-β inhibitor (Alk5i) or the described short-term rapamycin increased reprogramming efficiency, no further increase was observed by the combined treatment (Fig. 6D), which suggests an overlap in the action of both inhibitors. To more directly examine the relation between mTOR inhibition, senescence, and reprogramming, we knocked out p21^CIP1^. As shown previously, p21^CIP1^ knockout significantly increased reprogramming. Importantly, rapamycin treatment did not result in any additional increase, suggesting again that mTOR inhibition contributes to increase reprogramming efficiency by blunting senescence (Fig. 6D).

Together with previous findings, our results suggest a dual role for mTOR during reprogramming. While senescence is detrimental for reprogramming (Banito et al. 2009), the SASP favors reprogramming (Mosteiro et al. 2016), suggesting the coexistence of contrasting cell-intrinsic and cell-extrinsic effects. The scRNA-seq data showed that *MTOR* knockdown reduces SASP induction in response to OSKM expression (Fig. 4E,F). Previous work has shown that specific SASP components such as IL-6 can promote reprogramming (Mosteiro et al. 2016). The scRNA-seq showed that IL-6 was among several SASP components whose induction was reduced by mTOR inhibition (Fig. 4E), which we confirmed by quantitative RT–PCR (qRT–PCR) (Supplemental Fig. S7D). To understand whether mTOR inhibition could affect reprogramming by altering the SASP, we cultured TNG MKOS MEFs in the presence of different conditioned media (CMs). CM of senescent MEFs, but not that of MEFs in which *Mtor* expression was down-regulated, enhanced reprogramming efficiency (Fig. 6E; Supplemental Fig. S7E). Moreover, coculture of TNG MKOS MEFs with either control or senescent MEFs (expressing RAS^G12V^) or senescent MEFs infected with shMtor constructs rendered similar results (Supplemental Fig. S7F). Overall, the above experiments suggest that regulation of senescence by mTOR exerts opposing effects during cellular reprogramming (as summarized in Fig. 6F).

## Discussion

A better understanding of the mechanisms regulating cellular reprogramming is needed to increase its efficiency. Multiple efforts have been made to identify the different barriers that inherently limit cellular reprogramming (Qin et al. 2014; Sakurai et al. 2014; Yang et al. 2014). Senescence is one of these cell-intrinsic barriers (Banito et al. 2009). Despite the pathophysiological relevance of senescence, not much is known about how senescence is regulated during reprogramming beyond the involvement of the key tumor suppressors p53, p16^INK4a^, and p21^CIP1^ (Krizhanovsky and Lowe 2009; Banito and Gil 2010).

To gain further insight, we screened for shRNAs preventing OSKM-induced senescence. Our assay identified genes regulating OSKM-induced senescence without the confounding effects of reprogramming itself. We identified shRNAs targeting four genes: *CDKN1A*, *MTOR*, *MYOT*, and *UBE2E1*. While *CDKN1A* (which encodes for p21^CIP1^) and *MTOR* had been shown previously to regulate senescence or senescence-related phenotypes, this is the first evidence suggesting that *MYOT* and *UBE2E1* regulate senescence. *MYOT* encodes for myotilin, whose main known function is to be part of the Z-disc of sarcomeres (Otey et al. 2005). Despite identifying multiple shRNAs targeting *MYOT* in the screen and confirming their effects with two independent shRNAs, expression of *MYOT* was hard to detect in IMR90 cells. UBE2E1 is an E2 ubiquitin-conjugating enzyme (Nuber et al. 1996). Proteasome-dependent protein degradation had been observed previously during senescence (Deschenes-Simard et al. 2013). Whether UBE2E1 plays a role in that process will need to be studied. Given that the role of p21^CIP1^ in reprogramming and reprogramming-induced senescence is well known (Banito et al. 2009; Hong et al. 2009; Kawamura et al. 2009; Marion et al. 2009; Utikal et al. 2009), we decided to investigate the connection between mTOR, regulation of senescence, and reprogramming.

In addition, this screen provided us with a platform to test a new strategy to speed up candidate characterization by carrying out scRNA-seq in parallel with shRNA enrichment (Fig. 4A). First, we assessed the feasibility of detecting and assigning shRNAs to single cells. As we used a miR-30-based shRNA library (Fellmann et al. 2013), we were able to detect shRNA-specific sequence reads that could be distinguished from the endogenous mRNA sequence by filtering for reads that contained the unique shRNA hairpin sequence and part of the surrounding miR-30-based scaffold. We also proved that the detected shRNAs could be matched with their corresponding transcriptomes. Applying this methodology to our screen, we detected cells carrying shRNAs against two of the screen candidates (MTOR and CDKN1A). Due to the size of the library screened and the number of cells analyzed, we obtained only enough cells to infer information on *MTOR*. This limitation could be overcome by increasing the number of cells analyzed and/or decreasing the size of the library used for the screen. Recently, Perturb-seq and CRISP-seq, approaches that link CRISPR-based functional screens with scRNA-seq analysis, have been described (Adamson et al. 2016; Dixit et al. 2016; Jaitin et al. 2016). Although conceptually similar, the CRISPR-based approaches require the generation of specific vectors, including tags to monitor the expression of the single-guide RNAs (sgRNAs). As the cost of single-cell sequencing decreases, the approach described here could easily be scaled up, increasing the relevance of the information obtained. Our approach complements CRISPR-based technologies. For example, the knockdown achieved by shRNAs mimics the partial inhibition caused by drugs better than a CRISPR-mediated knockout. In addition, our approach could be used to analyze screens of microRNA (miRNA) libraries. Since it is believed that miRNAs can target multiple molecules in the same biological pathway, carrying out GO analysis of scRNA-seq in miRNA screens could be informative.

Our scRNA-seq analysis served to gain insights into the regulation of OSKM-induced senescence by mTOR and its impact on reprogramming. From the scRNA-seq, we obtained information on both genes and pathways affected by mTOR that served to investigate its mechanism of action. Two of the problems inherent to scRNA-seq are transcript detection and dropout rates per cell, which make it difficult to obtain significant results for individual lowly abundant genes. By extending our analysis to gene signatures instead, we increased the sensitivity of cell annotation, as the stochastic dropout is unlikely to affect all genes within a pathway at the same time, as shown previously (Mackenzie et al. 2017). Using this pathway-centric approach, we identified signatures affected by shMTOR from scRNA-seq, which allowed us to describe a role for TGF-β in regulating the expression of p21^CIP1^ and other CDKIs during OSKM-induced senescence.

Several reports have suggested that mTOR inhibition can enhance reprogramming (Chen et al. 2011; Wang et al. 2013). The effect is complex; low doses of mTOR inhibitors or short treatments increase the efficiency of reprogramming more than higher dosages or longer treatments (Chen et al. 2011). Until now, the experimental evidence focused only on autophagy to explain how mTOR regulates pluripotency and reprogramming (Wang et al. 2013; Wu et al. 2015). Our screen adds control of senescence as an additional mechanism. During oncogene-induced senescence (OIS), mTOR inhibition blunts SASP induction without preventing the senescent growth arrest (Herranz et al. 2015). In contrast, inhibiting mTOR during OSKM-induced senescence prevents both SASP induction and the senescent growth arrest. Since inhibiting the SASP decreased reprogramming efficiency, our study suggests an explanation for the dual antagonistic effects exerted by mTOR during reprogramming. The SASP comprises many factors, and while some of those factors favor reprogramming (e.g., IL6 or BMP family members), others can hinder it (such as TGFβ family members). Further dissecting which SASP components play positive or negative roles in reprogramming would be worth investigating.

Here, we identified mTOR as an important regulator of senescence during reprogramming. The net effect is explained in part by the contrasting effects exerted by mTOR over the senescence growth arrest and SASP induction. Besides clarifying the mechanism by which mTOR controls reprogramming-induced senescence, our work showcases the advantages of integrating scRNA-seq to accelerate the identification and characterization of candidate genes in functional screens.

## Materials and methods

### Cell culture

IMR90 human fetal lung fibroblasts (American Type Culture Collection) were cultured in high-glucose Dulbecco's modified Eagle medium (DMEM) (Gibco, 41965) supplemented with 10% fetal bovine serum (FBS) (Sigma) and 1× antibiotic–anti-mycotic (Gibco, 15240) and grown at 37°C and 5% CO_2_. TNG MKOS MEFs containing a dox-inducible MKOS-IRES-mOrange transgene and a Nanog-GFP reporter were isolated from embryonic day 12.5 (E12.5) chimeric embryos with a 129 genetic background as described before (O'Malley et al. 2013). Wild-type MEFs were prepared from 13.5-d-old embryos of CD-1 mice (Tordella et al. 2016). MEFs were maintained in MEF medium (Glasgow minimum essential medium [GMEM]; Sigma, G5154) supplemented with 10% EmbryoMax ES cell qualified FBS (Millipore, ES-009-B), 2 mM L-glutamine (Gibco, 25030), 1× MEM nonessential amino acids (NEAA) (Gibco, 11140), 1 mM sodium pyruvate (Gibco, 11360), 1× antibiotic–anti-mycotic (Gibco, 15240), 50 µM 2-mercaptoethanol (Gibco, 31350) supplemented with 1 ng/mL heparin (Sigma, H3149), and 5 ng/mL recombinant human FGF-basic (AA 1-155; Gibco, PHG0264).

### Plasmids

For de novo generation of miRE-based shRNAs, 97-mer oligonucleotides containing the shRNA fragment were PCR-amplified using primers miRE-Xho-fw and miRE-EcoOligo-rev and cloned into the pRLL lentiviral backbone as before (Fellmann et al. 2013). shRNA sequences are listed in Supplemental Table S1.

### shRNA libraries and screening

Details of the shRNA libraries used and the screen protocol are in the Supplemental Material.

### Library preparation for determining shRNA enrichment

Genomic DNA was extracted from 10^6^ cells collected at different time points during the screen using the Gentra Puregene cell kit (Qiagen) as described by the manufacturer. Solexa adapters and a sample-specific barcode (3 nucleotides [nt] for pGIPZ and 4 nt for pRLL), to allow for multiplexing, were ligated by PCR using 2 μg of extracted DNA as a template. Individual barcoded PCR products were purified using the QIAquick gel extraction kit (Qiagen), quantified using the Qubit 2.0 Fluorometer, and pooled by combining equal quantities (40 ng each). Prior to sequencing, the resulting Solexa library was quantified using qPCR and the Bioanalyzer Agilent high-sensitivity DNA kit (Agilent Technologies) to estimate the average size of the library. Of the library, 12 pM was applied to an Illumina flow cell for cluster generation and sequenced on an Illumina Genome Analyzer IIx system or HiSeq 2500 in rapid-run mode (50-base-pair single read), following the manufacturers’ protocols.

### Statistical analysis of the shRNA screens

FASTA files produced from the sequencing runs were processed, and sequences were demultiplexed with CASAVA version 1.8. The reverse complement of each read was aligned to the custom shRNA libraries using Bowtie (version 0.12.8). Candidates were ranked using Fisher's combined *P*-value and edgeR. The Fisher's combined test allowed *P*-values across independent data sets to be combined, bearing on the same overall hypothesis (Fisher 1925).

### Immunofluorescence and high-content analysis

Cells were fixed in 4% formaldehyde for at least 30 min, washed with PBS, and permeabilized with 0.2% Triton X-100 for 5 min. After 30 min in blocking solution (BS; 0.5% BSA, 0.2% fish skin gelatin in PBS), cells were incubated with the primary antibody diluted in BS for 1 h at room temperature. Cells were washed three times with PBS and incubated with Alexa fluor-labeled secondary antibodies for 30 min at room temperature. After three washes with PBS, nuclei were stained with 1 µg/mL DAPI for 10 min. The antibodies used are described in Supplemental Table S2. The high-content analysis is described in detail in the Supplemental Material.

### BrdU incorporation, SA-β-Gal, and crystal violet staining

BrdU incorporation, SA-β-Gal, and crystal violet staining assays are described in the Supplemental Material.

### mRNA expression analysis

Total RNA was extracted using Trizol reagent (Invitrogen) per the manufacturer's instructions. cDNA was generated using random hexamers and SuperScript II reverse transcriptase (Invitrogen). Quantitative real-time PCR was performed using SYBR Green PCR master mix (Applied Biosystems) in a CFX96 real-time PCR detection system (Bio-Rad). Primers are listed in Supplemental Table S3. *GAPDH* expression was used for normalization.

### Preparation of libraries for scRNA-seq and scRNA-seq data analysis

Detailed protocols describing the preparation of Smart-Seq2 and ICELL8 single-cell libraries as well as scRNA-seq data analysis are in the Supplemental Material.

### Reprogramming experiments with TNG MKOS MEFs

TNG MKOS MEFs were obtained via morula aggregation as described previously (Chantzoura et al. 2015). A detailed description of the reprogramming protocol is in the Supplemental Material.

### CM

To produce CM, MEFs were infected with RAS and pooled shRNAs against *Mtor* or control vectors and cultured in selection medium for 11 d, after which the medium was changed to MEF medium with 0.5% FBS for conditioning. CM was collected and filtered (0.22 µm) after 3 d in two consecutive rounds. CM aliquots were stored at −20°C. For the reprogramming assays, CM was mixed with 4× concentrated reprogramming medium in a proportion of 3 to 1.

### Coculture experiments

Approximately 2 × 10^5^ MEFs infected with RAS-expressing or vector control retroviruses were seeded per gelatinized six-well plate. The next day, 2 × 10^3^ TNG MKOS MEFs were seeded on top.

### Statistical analysis

Statistical analysis of data was conducted using Graphpad Prism 7 software. Unpaired Student's *t*-test was used to calculate *P*-values.

### Accession numbers

RNA-seq data sets have been deposited at the Gene Expression Omnibus (accession no. GSE95021).

## Supplementary Material

Supplemental Material

## References

[AARTSGAD297796C1] Acosta JC, O'Loghlen A, Banito A, Guijarro MV, Augert A, Raguz S, Fumagalli M, Da Costa M, Brown C, Popov N, 2008 Chemokine signaling via the CXCR2 receptor reinforces senescence. Cell 133: 1006–1018. 18555777 10.1016/j.cell.2008.03.038

[AARTSGAD297796C2] Adamson B, Norman TM, Jost M, Cho MY, Nunez JK, Chen Y, Villalta JE, Gilbert LA, Horlbeck MA, Hein MY, 2016 A multiplexed single-cell CRISPR screening platform enables systematic dissection of the unfolded protein response. Cell 167: 1867–1882 e1821. 27984733 10.1016/j.cell.2016.11.048 PMC5315571

[AARTSGAD297796C3] Baker DJ, Childs BG, Durik M, Wijers ME, Sieben CJ, Zhong J, Saltness RA, Jeganathan KB, Verzosa GC, Pezeshki A, 2016 Naturally occurring p16(Ink4a)-positive cells shorten healthy lifespan. Nature 530: 184–189. 26840489 10.1038/nature16932 PMC4845101

[AARTSGAD297796C4] Banito A, Gil J. 2010 Induced pluripotent stem cells and senescence: learning the biology to improve the technology. EMBO Rep 11: 353–359. 20379220 10.1038/embor.2010.47 PMC2868548

[AARTSGAD297796C5] Banito A, Rashid ST, Acosta JC, Li S, Pereira CF, Geti I, Pinho S, Silva JC, Azuara V, Walsh M, 2009 Senescence impairs successful reprogramming to pluripotent stem cells. Genes Dev 23: 2134–2139. 19696146 10.1101/gad.1811609 PMC2751980

[AARTSGAD297796C6] Brady JJ, Li M, Suthram S, Jiang H, Wong WH, Blau HM. 2013 Early role for IL-6 signalling during generation of induced pluripotent stem cells revealed by heterokaryon RNA-seq. Nat Cell Biol 15: 1244–1252. 23995732 10.1038/ncb2835 PMC4100556

[AARTSGAD297796C7] Chang HY, Sneddon JB, Alizadeh AA, Sood R, West RB, Montgomery K, Chi JT, van de Rijn M, Botstein D, Brown PO. 2004 Gene expression signature of fibroblast serum response predicts human cancer progression: similarities between tumors and wounds. PLoS Biol 2: E7. 14737219 10.1371/journal.pbio.0020007 PMC314300

[AARTSGAD297796C8] Chantzoura E, Skylaki S, Menendez S, Kim SI, Johnsson A, Linnarsson S, Woltjen K, Chambers I, Kaji K. 2015 Reprogramming roadblocks are system dependent. Stem Cell Reports 5: 350–364. 26278041 10.1016/j.stemcr.2015.07.007 PMC4618455

[AARTSGAD297796C9] Cheloufi S, Elling U, Hopfgartner B, Jung YL, Murn J, Ninova M, Hubmann M, Badeaux AI, Euong Ang C, Tenen D, 2015 The histone chaperone CAF-1 safeguards somatic cell identity. Nature 528: 218–224. 26659182 10.1038/nature15749 PMC4866648

[AARTSGAD297796C10] Chen T, Shen L, Yu J, Wan H, Guo A, Chen J, Long Y, Zhao J, Pei G. 2011 Rapamycin and other longevity-promoting compounds enhance the generation of mouse induced pluripotent stem cells. Aging Cell 10: 908–911. 21615676 10.1111/j.1474-9726.2011.00722.x

[AARTSGAD297796C11] Collado M, Serrano M. 2010 Senescence in tumours: evidence from mice and humans. Nat Rev Cancer 10: 51–57. 20029423 10.1038/nrc2772 PMC3672965

[AARTSGAD297796C12] Coppe JP, Desprez PY, Krtolica A, Campisi J. 2010 The senescence-associated secretory phenotype: the dark side of tumor suppression. Annu Rev Pathol 5: 99–118. 20078217 10.1146/annurev-pathol-121808-102144 PMC4166495

[AARTSGAD297796C13] Deschenes-Simard X, Gaumont-Leclerc MF, Bourdeau V, Lessard F, Moiseeva O, Forest V, Igelmann S, Mallette FA, Saba-El-Leil MK, Meloche S, 2013 Tumor suppressor activity of the ERK/MAPK pathway by promoting selective protein degradation. Genes Dev 27: 900–915. 23599344 10.1101/gad.203984.112 PMC3650227

[AARTSGAD297796C14] Dickins RA, Hemann MT, Zilfou JT, Simpson DR, Ibarra I, Hannon GJ, Lowe SW. 2005 Probing tumor phenotypes using stable and regulated synthetic microRNA precursors. Nat Genet 37: 1289–1295. 16200064 10.1038/ng1651

[AARTSGAD297796C15] Dixit A, Parnas O, Li B, Chen J, Fulco CP, Jerby-Arnon L, Marjanovic ND, Dionne D, Burks T, Raychowdhury R, 2016 Perturb-seq: dissecting molecular circuits with scalable single-cell RNA profiling of pooled genetic screens. Cell 167: 1853–1866.e17. 27984732 10.1016/j.cell.2016.11.038 PMC5181115

[AARTSGAD297796C16] Fellmann C, Hoffmann T, Sridhar V, Hopfgartner B, Muhar M, Roth M, Lai DY, Barbosa IA, Kwon JS, Guan Y, 2013 An optimized microRNA backbone for effective single-copy RNAi. Cell Rep 5: 1704–1713. 24332856 10.1016/j.celrep.2013.11.020

[AARTSGAD297796C17] Fisher RA. 1925 The resemblance between twins, a statistical examination of Lauterbach's measurements. Genetics 10: 569–579. 17246289 10.1093/genetics/10.6.569 PMC1200877

[AARTSGAD297796C18] Gurdon JB. 1962 Adult frogs derived from the nuclei of single somatic cells. Dev Biol 4: 256–273. 13903027 10.1016/0012-1606(62)90043-x

[AARTSGAD297796C19] Herranz N, Gallage S, Mellone M, Wuestefeld T, Klotz S, Hanley CJ, Raguz S, Acosta JC, Innes AJ, Banito A, 2015 mTOR regulates MAPKAPK2 translation to control the senescence-associated secretory phenotype. Nat Cell Biol 17: 1205–1217. 26280535 10.1038/ncb3225 PMC4589897

[AARTSGAD297796C20] Hong H, Takahashi K, Ichisaka T, Aoi T, Kanagawa O, Nakagawa M, Okita K, Yamanaka S. 2009 Suppression of induced pluripotent stem cell generation by the p53–p21 pathway. Nature 460: 1132–1135. 19668191 10.1038/nature08235 PMC2917235

[AARTSGAD297796C21] Jacobs JJ, Keblusek P, Robanus-Maandag E, Kristel P, Lingbeek M, Nederlof PM, van Welsem T, van de Vijver MJ, Koh EY, Daley GQ, 2000 Senescence bypass screen identifies TBX2, which represses Cdkn2a (p19(ARF)) and is amplified in a subset of human breast cancers. Nat Genet 26: 291–299. 11062467 10.1038/81583

[AARTSGAD297796C22] Jaitin DA, Weiner A, Yofe I, Lara-Astiaso D, Keren-Shaul H, David E, Salame TM, Tanay A, van Oudenaarden A, Amit I. 2016 Dissecting immune circuits by linking CRISPR-pooled screens with single-cell RNA-seq. Cell 167: 1883–1896.e15. 27984734 10.1016/j.cell.2016.11.039

[AARTSGAD297796C23] Kawamura T, Suzuki J, Wang YV, Menendez S, Morera LB, Raya A, Wahl GM, Belmonte JC. 2009 Linking the p53 tumour suppressor pathway to somatic cell reprogramming. Nature 460: 1140–1144. 19668186 10.1038/nature08311 PMC2735889

[AARTSGAD297796C24] Krizhanovsky V, Lowe SW. 2009 Stem cells: the promises and perils of p53. Nature 460: 1085–1086. 19713919 10.1038/4601085a PMC2974062

[AARTSGAD297796C25] Krizhanovsky V, Yon M, Dickins RA, Hearn S, Simon J, Miething C, Yee H, Zender L, Lowe SW. 2008 Senescence of activated stellate cells limits liver fibrosis. Cell 134: 657–667. 18724938 10.1016/j.cell.2008.06.049 PMC3073300

[AARTSGAD297796C26] Kuilman T, Michaloglou C, Mooi WJ, Peeper DS. 2010 The essence of senescence. Genes Dev 24: 2463–2479. 21078816 10.1101/gad.1971610 PMC2975923

[AARTSGAD297796C27] Li H, Collado M, Villasante A, Strati K, Ortega S, Canamero M, Blasco MA, Serrano M. 2009 The Ink4/Arf locus is a barrier for iPS cell reprogramming. Nature 460: 1136–1139. 19668188 10.1038/nature08290 PMC3578184

[AARTSGAD297796C28] Mackenzie KJ, Carroll P, Martin CA, Murina O, Fluteau A, Simpson DJ, Olova N, Sutcliffe H, Rainger JK, Leitch A, 2017 cGAS surveillance of micronuclei links genome instability to innate immunity. Nature 548: 461–465. 28738408 10.1038/nature23449 PMC5870830

[AARTSGAD297796C29] Marion RM, Strati K, Li H, Murga M, Blanco R, Ortega S, Fernandez-Capetillo O, Serrano M, Blasco MA. 2009 A p53-mediated DNA damage response limits reprogramming to ensure iPS cell genomic integrity. Nature 460: 1149–1153. 19668189 10.1038/nature08287 PMC3624089

[AARTSGAD297796C30] Mosteiro L, Pantoja C, Alcazar N, Marion RM, Chondronasiou D, Rovira M, Fernandez-Marcos PJ, Munoz-Martin M, Blanco-Aparicio C, Pastor J, 2016 Tissue damage and senescence provide critical signals for cellular reprogramming in vivo. Science 354: aaf4445. 27884981 10.1126/science.aaf4445

[AARTSGAD297796C31] Munoz-Espin D, Serrano M. 2014 Cellular senescence: from physiology to pathology. Nat Rev Mol Cell Biol 15: 482–496. 24954210 10.1038/nrm3823

[AARTSGAD297796C32] Nuber U, Schwarz S, Kaiser P, Schneider R, Scheffner M. 1996 Cloning of human ubiquitin-conjugating enzymes UbcH6 and UbcH7 (E2-F1) and characterization of their interaction with E6-AP and RSP5. J Biol Chem 271: 2795–2800. 8576257 10.1074/jbc.271.5.2795

[AARTSGAD297796C33] O'Malley J, Skylaki S, Iwabuchi KA, Chantzoura E, Ruetz T, Johnsson A, Tomlinson SR, Linnarsson S, Kaji K. 2013 High-resolution analysis with novel cell-surface markers identifies routes to iPS cells. Nature 499: 88–91. 23728301 10.1038/nature12243 PMC3743022

[AARTSGAD297796C34] Otey CA, Rachlin A, Moza M, Arneman D, Carpen O. 2005 The palladin/myotilin/myopalladin family of actin-associated scaffolds. Int Rev Cytol 246: 31–58. 16164966 10.1016/S0074-7696(05)46002-7

[AARTSGAD297796C35] Picelli S, Faridani OR, Bjorklund AK, Winberg G, Sagasser S, Sandberg R. 2014 Full-length RNA-seq from single cells using Smart-seq2. Nat Protoc 9: 171–181. 24385147 10.1038/nprot.2014.006

[AARTSGAD297796C36] Qin H, Diaz A, Blouin L, Lebbink RJ, Patena W, Tanbun P, LeProust EM, McManus MT, Song JS, Ramalho-Santos M. 2014 Systematic identification of barriers to human iPSC generation. Cell 158: 449–461. 25036638 10.1016/j.cell.2014.05.040 PMC4130998

[AARTSGAD297796C37] Reynisdottir I, Polyak K, Iavarone A, Massague J. 1995 Kip/Cip and Ink4 Cdk inhibitors cooperate to induce cell cycle arrest in response to TGF-β. Genes Dev 9: 1831–1845. 7649471 10.1101/gad.9.15.1831

[AARTSGAD297796C38] Rowland BD, Bernards R, Peeper DS. 2005 The KLF4 tumour suppressor is a transcriptional repressor of p53 that acts as a context-dependent oncogene. Nat Cell Biol 7: 1074–1082. 16244670 10.1038/ncb1314

[AARTSGAD297796C39] Sakurai K, Talukdar I, Patil VS, Dang J, Li Z, Chang KY, Lu CC, Delorme-Walker V, Dermardirossian C, Anderson K, 2014 Kinome-wide functional analysis highlights the role of cytoskeletal remodeling in somatic cell reprogramming. Cell Stem Cell 14: 523–534. 24702998 10.1016/j.stem.2014.03.001 PMC4071169

[AARTSGAD297796C40] Salama R, Sadaie M, Hoare M, Narita M. 2014 Cellular senescence and its effector programs. Genes Dev 28: 99–114. 24449267 10.1101/gad.235184.113 PMC3909793

[AARTSGAD297796C41] Shi Y, Inoue H, Wu JC, Yamanaka S. 2017 Induced pluripotent stem cell technology: a decade of progress. Nat Rev Drug Discov 16: 115–130. 27980341 10.1038/nrd.2016.245 PMC6416143

[AARTSGAD297796C42] Tada M, Takahama Y, Abe K, Nakatsuji N, Tada T. 2001 Nuclear reprogramming of somatic cells by in vitro hybridization with ES cells. Curr Biol 11: 1553–1558. 11591326 10.1016/s0960-9822(01)00459-6

[AARTSGAD297796C43] Takahashi K, Yamanaka S. 2006 Induction of pluripotent stem cells from mouse embryonic and adult fibroblast cultures by defined factors. Cell 126: 663–676. 16904174 10.1016/j.cell.2006.07.024

[AARTSGAD297796C44] Tanay A, Regev A. 2017 Scaling single-cell genomics from phenomenology to mechanism. Nature 541: 331–338. 28102262 10.1038/nature21350 PMC5438464

[AARTSGAD297796C45] Tordella L, Khan S, Hohmeyer A, Banito A, Klotz S, Raguz S, Martin N, Dhamarlingam G, Carroll T, Gonzalez Meljem JM, 2016 SWI/SNF regulates a transcriptional program that induces senescence to prevent liver cancer. Genes Dev 30: 2187–2198. 27737960 10.1101/gad.286112.116 PMC5088567

[AARTSGAD297796C46] Tzelepis K, Koike-Yusa H, De Braekeleer E, Li Y, Metzakopian E, Dovey OM, Mupo A, Grinkevich V, Li M, Mazan M, 2016 A CRISPR dropout screen identifies genetic vulnerabilities and therapeutic targets in acute myeloid leukemia. Cell Rep 17: 1193–1205. 27760321 10.1016/j.celrep.2016.09.079 PMC5081405

[AARTSGAD297796C47] Utikal J, Polo JM, Stadtfeld M, Maherali N, Kulalert W, Walsh RM, Khalil A, Rheinwald JG, Hochedlinger K. 2009 Immortalization eliminates a roadblock during cellular reprogramming into iPS cells. Nature 460: 1145–1148. 19668190 10.1038/nature08285 PMC3987892

[AARTSGAD297796C48] Vijayachandra K, Higgins W, Lee J, Glick A. 2009 Induction of p16ink4a and p19ARF by TGFβ1 contributes to growth arrest and senescence response in mouse keratinocytes. Mol Carcinog 48: 181–186. 18655107 10.1002/mc.20472

[AARTSGAD297796C49] Wang S, Xia P, Ye B, Huang G, Liu J, Fan Z. 2013 Transient activation of autophagy via Sox2-mediated suppression of mTOR is an important early step in reprogramming to pluripotency. Cell Stem Cell 13: 617–625. 24209762 10.1016/j.stem.2013.10.005

[AARTSGAD297796C50] Wang Y, Xu Q, Sack L, Kang C, Elledge SJ. 2016 A gain-of-function senescence bypass screen identifies the homeobox transcription factor DLX2 as a regulator of ATM–p53 signaling. Genes Dev 30: 293–306. 26833729 10.1101/gad.271445.115 PMC4743059

[AARTSGAD297796C51] Wilmut I, Schnieke AE, McWhir J, Kind AJ, Campbell KH. 1997 Viable offspring derived from fetal and adult mammalian cells. Nature 385: 810–813. 9039911 10.1038/385810a0

[AARTSGAD297796C52] Wu Y, Li Y, Zhang H, Huang Y, Zhao P, Tang Y, Qiu X, Ying Y, Li W, Ni S, 2015 Autophagy and mTORC1 regulate the stochastic phase of somatic cell reprogramming. Nat Cell Biol 17: 715–725. 25985393 10.1038/ncb3172

[AARTSGAD297796C53] Yang CS, Chang KY, Rana TM. 2014 Genome-wide functional analysis reveals factors needed at the transition steps of induced reprogramming. Cell Rep 8: 327–337. 25043178 10.1016/j.celrep.2014.07.002 PMC4152236

