# Supplementary material for: Coupling shRNA screens with single-cell RNA-seq identifies a dual role for mTOR in reprogramming-induced senescence
Source: Genes Dev. 2017 Oct 15;31(20):2085–98. doi: 10.1101/gad.297796.117 (PMC5733499; doi:10.1101/gad.297796.117)
Supplement: Supplemental Material [file supp_31_20_2085__index.html]

Coupling shRNA screens with single-cell RNA-seq identifies a dual role for mTOR in reprogramming-induced senescence — Supplemental Material 

# Coupling shRNA screens with single-cell RNA-seq identifies a dual role for mTOR in reprogramming-induced senescence

## Supplemental Material

- Supplemental\_Information.pdf
