# Supplementary material for: Coupling shRNA screens with single-cell RNA-seq identifies a dual role for mTOR in reprogramming-induced senescence
Source: Genes Dev. 2017 Oct 15;31(20):2085–98. doi: 10.1101/gad.297796.117 (PMC5733499; doi:10.1101/gad.297796.117)
Supplement: Supplemental Material [file supp_gad.297796.117_Supplemental_Information.pdf]

SUPPLEMENTAL DATA for

Coupling shRNA screens with single-cell RNA-Seq identifies a dual role for mTOR in reprogramming-induced senescence

Marieke Aarts, Athena Georgilis, Meryam Beniazza, Patrizia Beolchi, Ana Banito, Thomas Carroll, Marizela Kulisic, Daniel F. Kaemena, Gopuraja Dharmalingam, Nadine Martin, Wolf Reik, Johannes Zuber, Keisuke Kaji, Tamir Chandra and Jesús Gil

Including:

- **Supplemental Figures S1 to S7 and legends**
- **Supplemental Tables S1 to S4**
- **Supplemental Materials and Methods**
- **Supplemental References**

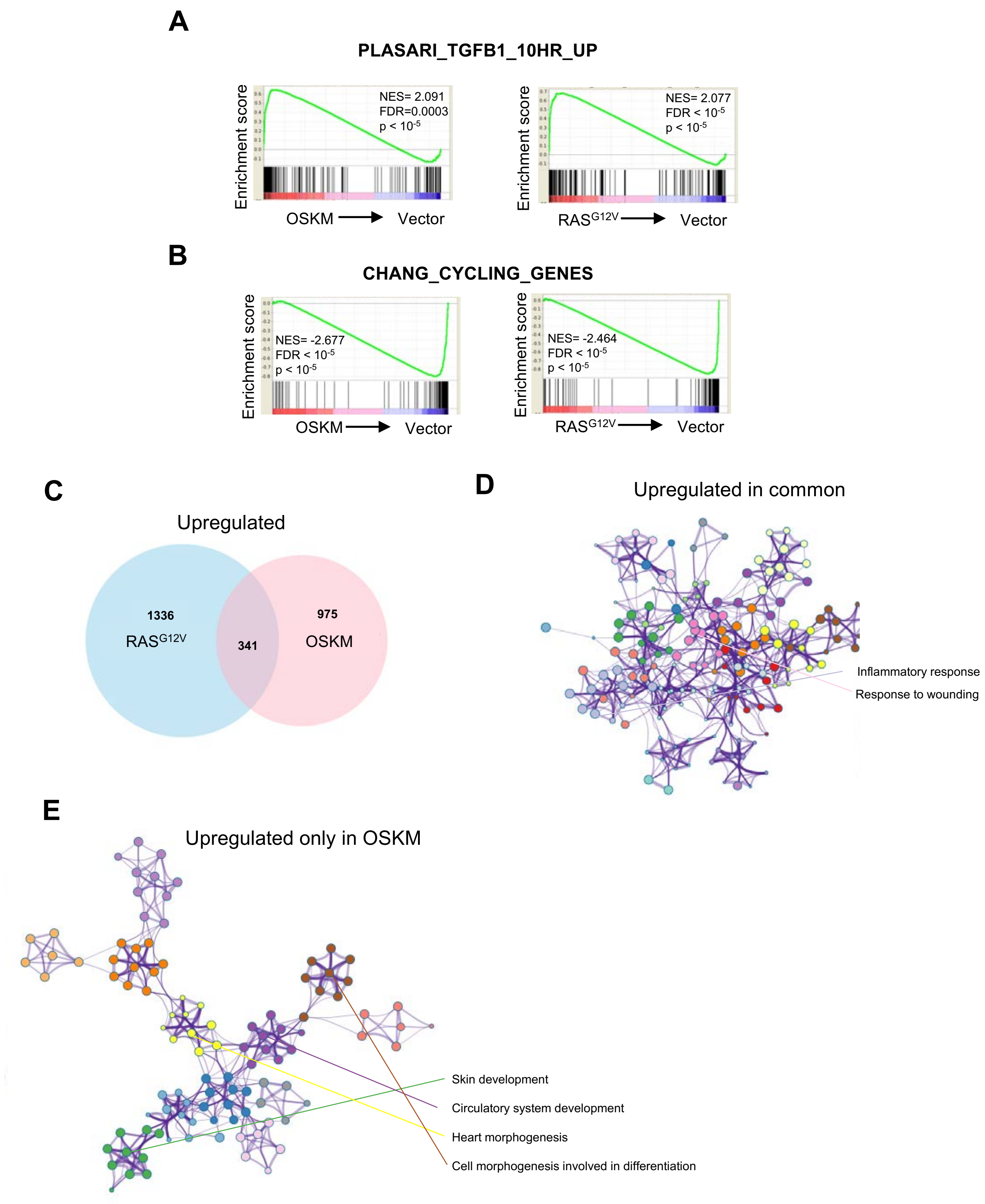
A
PLASARI_TGFB1_10HR_UP
Enrichment score
NES= 2.091
FDR=0.0003
p < 10-5
OSKM → Vector
NES= 2.077
FDR < 10-5
p < 10-5
RASG12V → Vector
B
CHANG_CYCLING_GENES
NES= -2.677
FDR < 10-5
p < 10-5
NES= -2.464
FDR < 10-5
p < 10-5
C
Upregulated
1336
RASG12V
341
975
OSKM
D
Upregulated in common
Inflammatory response
Response to wounding
E
Upregulated only in OSKM
Skin development
Circulatory system development
Heart morphogenesis
Cell morphogenesis involved in differentiation

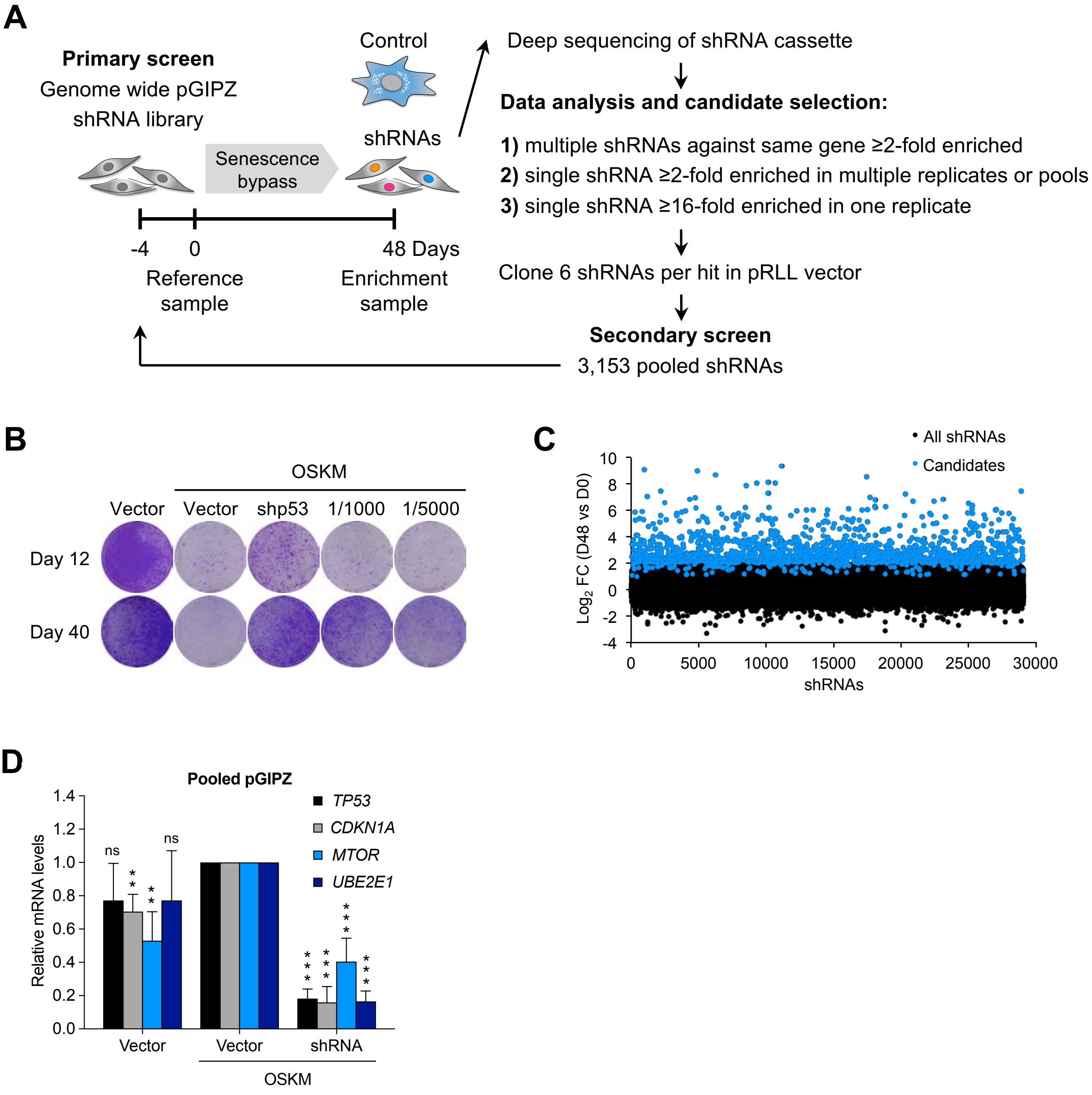
A
Primary screen
Genome wide pGIPZ
shRNA library
Senescence
bypass
Control
shRNAs
-4
0
48 Days
Reference
sample
Enrichment
sample
Deep sequencing of shRNA cassette
Data analysis and candidate selection:
1) multiple shRNAs against same gene ≥2-fold enriched
2) single shRNA ≥2-fold enriched in multiple replicates or pools
3) single shRNA ≥16-fold enriched in one replicate
Clone 6 shRNAs per hit in pRLL vector
Secondary screen
3,153 pooled shRNAs
B
OSKM
Vector
Vector
shp53
1/1000
1/5000
Day 12
Day 40
C
All shRNAs
Candidates
Log2 FC (D48 vs D0)
shRNAs
D
Pooled pGIPZ
TP53
CDKN1A
MTOR
UBE2E1
Relative mRNA levels
ns
Vector
Vector
shRNA
OSKM

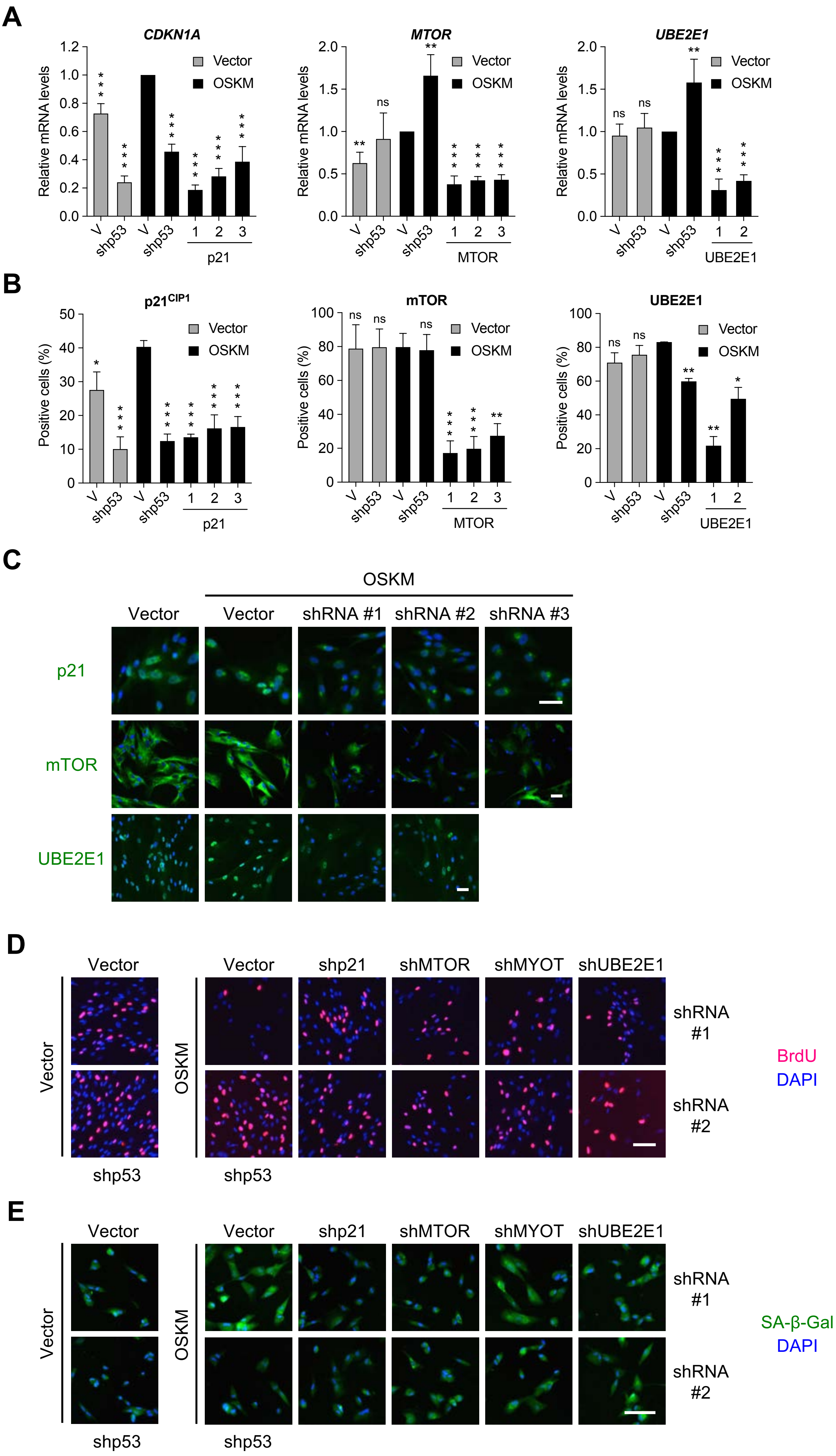
A
CDKN1A
MTOR
UBE2E1
Relative mRNA levels
Vector
OSKM
V
shp53
p21
MTOR
UBE2E1
B
p21CIP1
mTOR
UBE2E1
Positive cells (%)
C
OSKM
Vector
shRNA #1
shRNA #2
shRNA #3
p21
mTOR
UBE2E1
D
Vector
shp21
shMTOR
shMYOT
shUBE2E1
OSKM
shRNA #1
shRNA #2
BrdU
DAPI
shp53
E
SA-β-Gal
DAPI

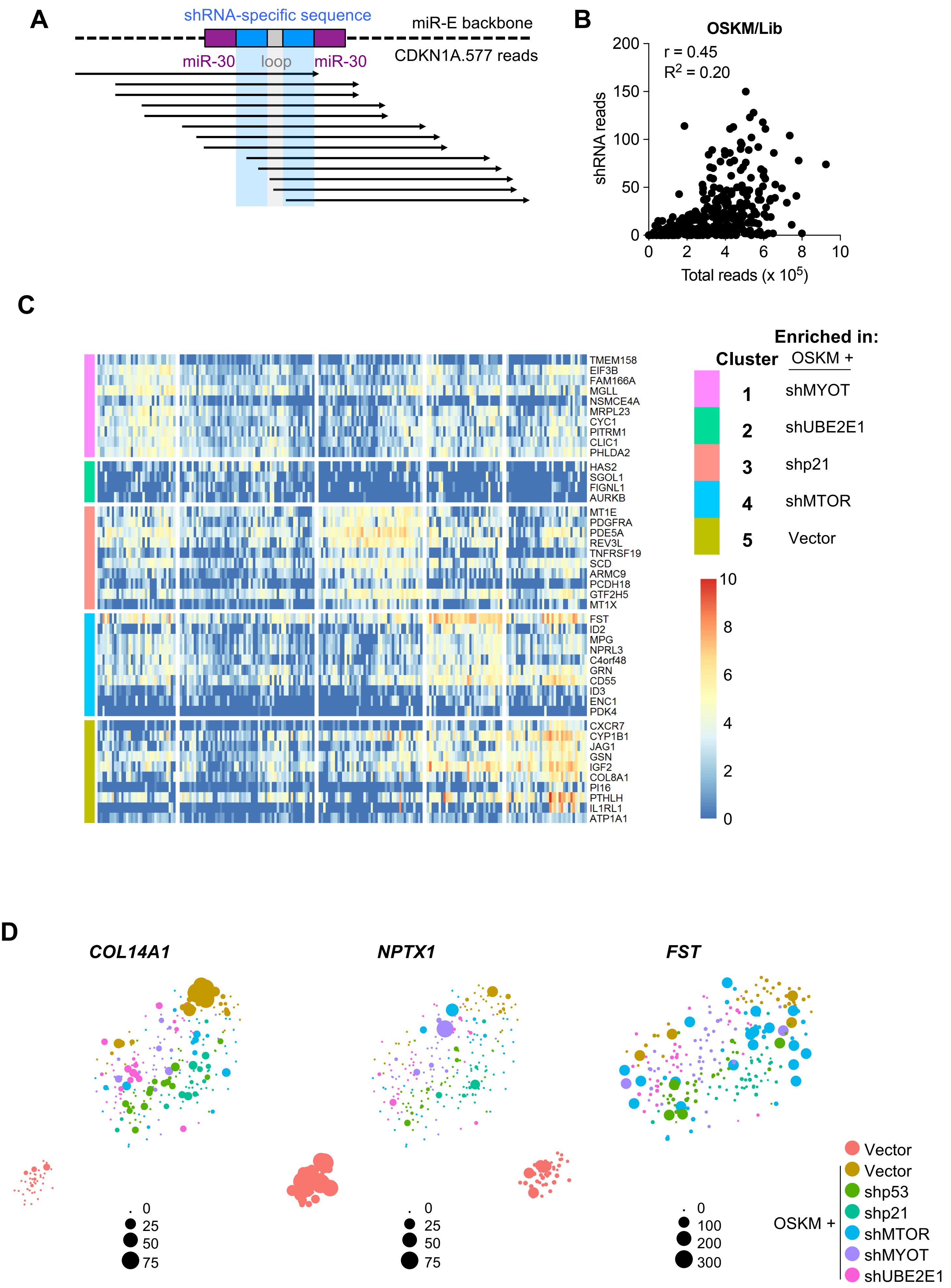
A
shRNA-specific sequence
miR-E backbone
miR-30
loop
miR-30
CDKN1A.577 reads
B
OSKM/Lib
r = 0.45
R2 = 0.20
shRNA reads
Total reads (x 105)
C
Enriched in:
Cluster
OSKM +
1 shMYOT
2 shUBE2E1
3 shp21
4 shMTOR
5 Vector
TMEM158
EIF3B
FAM166A
MGLL
NSMCE4A
MRPL23
CYC1
PITRM1
CLIC1
PHLDA2
HAS2
SGOL1
FIGNL1
AURKB
MT1E
PDGFRA
PDE5A
REV3L
TNFRSF19
SCD
ARMC9
PCDH18
GTF2H5
MT1X
FST
ID2
MPG
NPRL3
C4orf48
GRN
CD55
ID3
ENC1
PDK4
CXCR7
CYP1B1
JAG1
GSN
IGF2
COL8A1
PI16
PTHLH
IL1RL1
ATP1A1
D
COL14A1
NPTX1
FST
Vector
OSKM +
Vector
shp53
shp21
shMTOR
shMYOT
shUBE2E1

Aarts_**Sup. Figure 5**

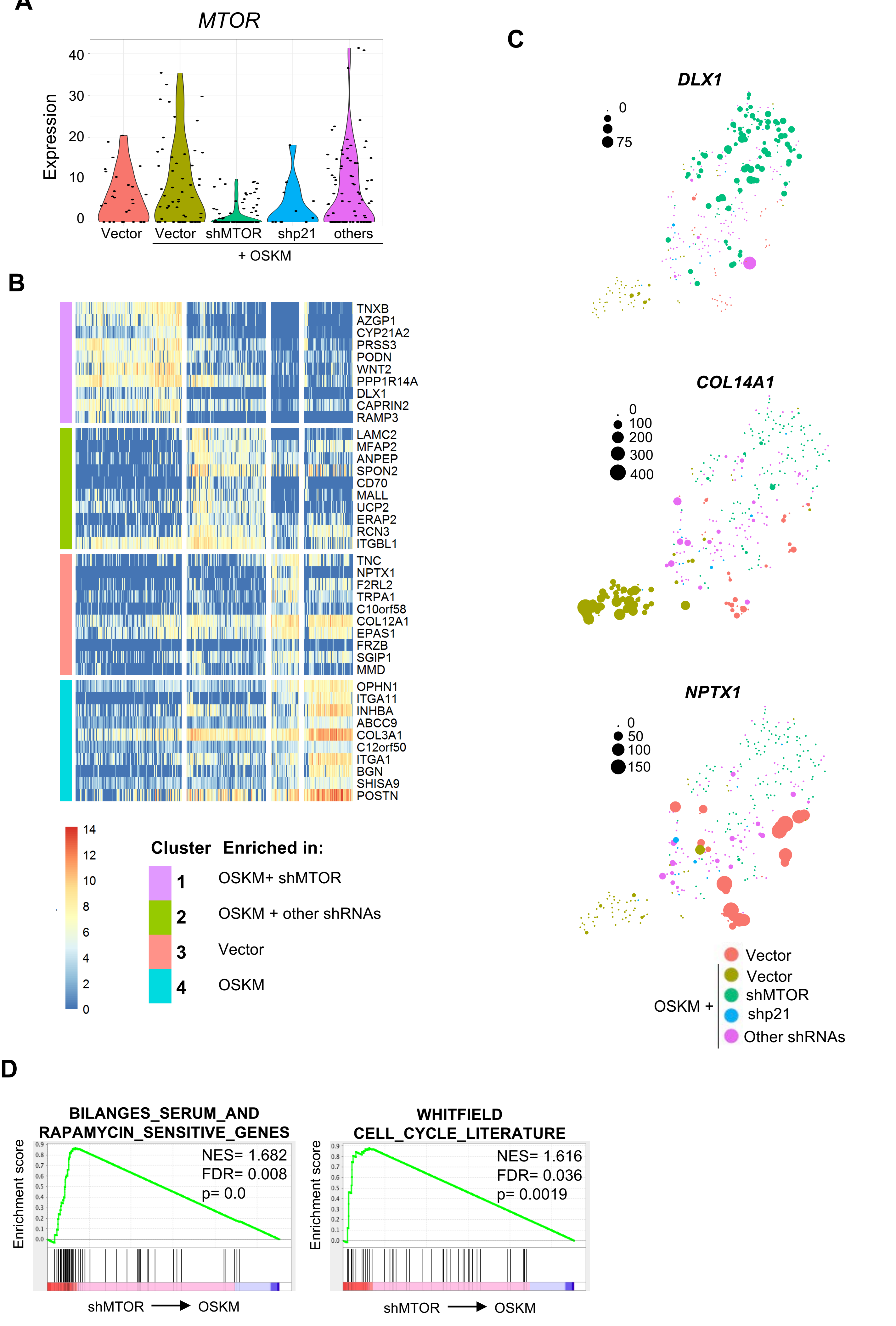

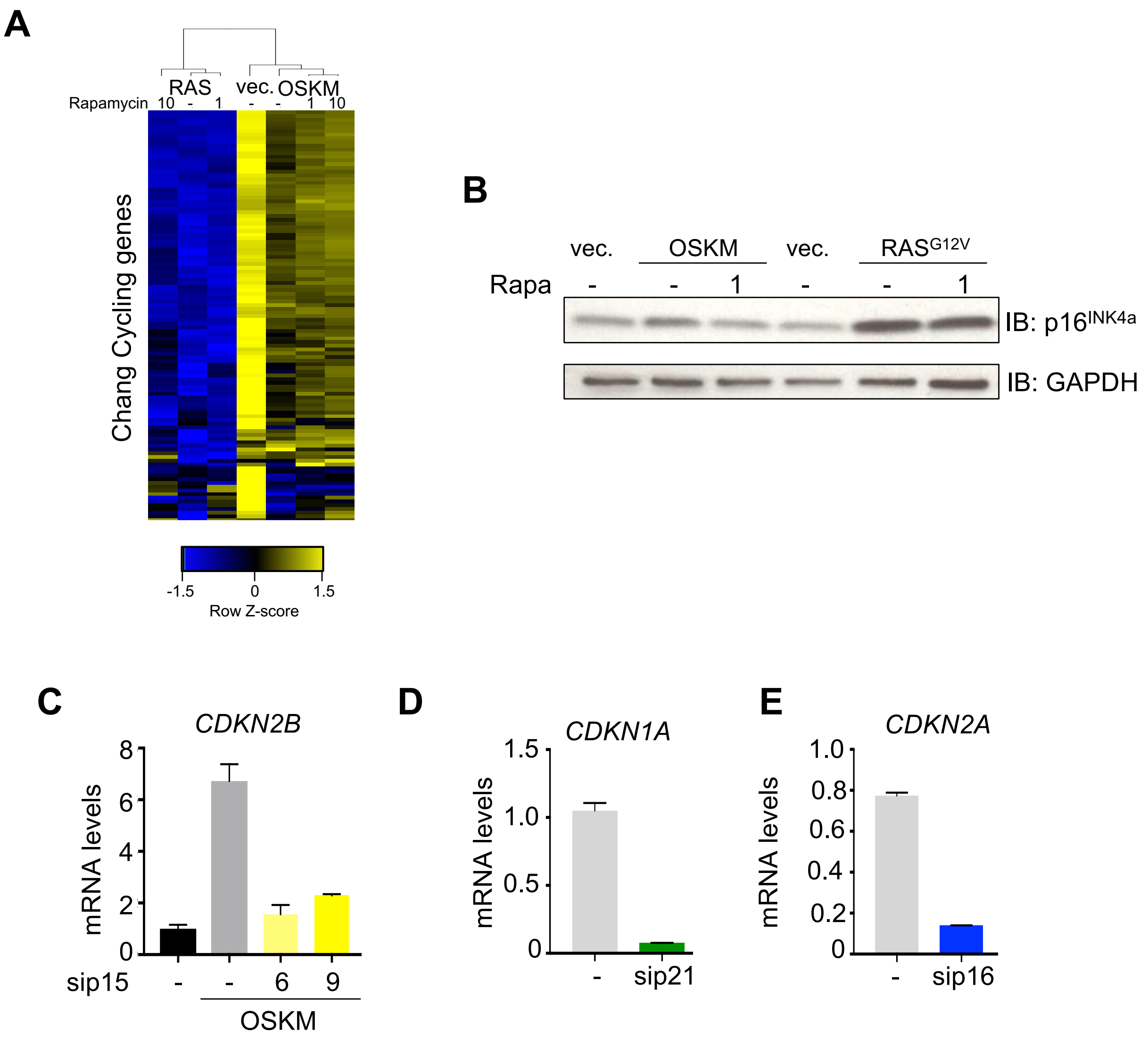
A
RAS
vec.
OSKM
Rapamycin 10 - 1 - - 1 10
Chang Cycling genes
-1.5
0
1.5
Row Z-score
B
vec.
OSKM
vec.
RASG12V
Rapa - - 1 - - 1
IB: p16INK4a
IB: GAPDH
C
CDKN2B
mRNA levels
8
6
4
2
0
sip15 - - 6 9
OSKM
D
CDKN1A
mRNA levels
1.5
1.0
0.5
0
- sip21
E
CDKN2A
mRNA levels
1.0
0.8
0.6
0.4
0.2
0
- sip16

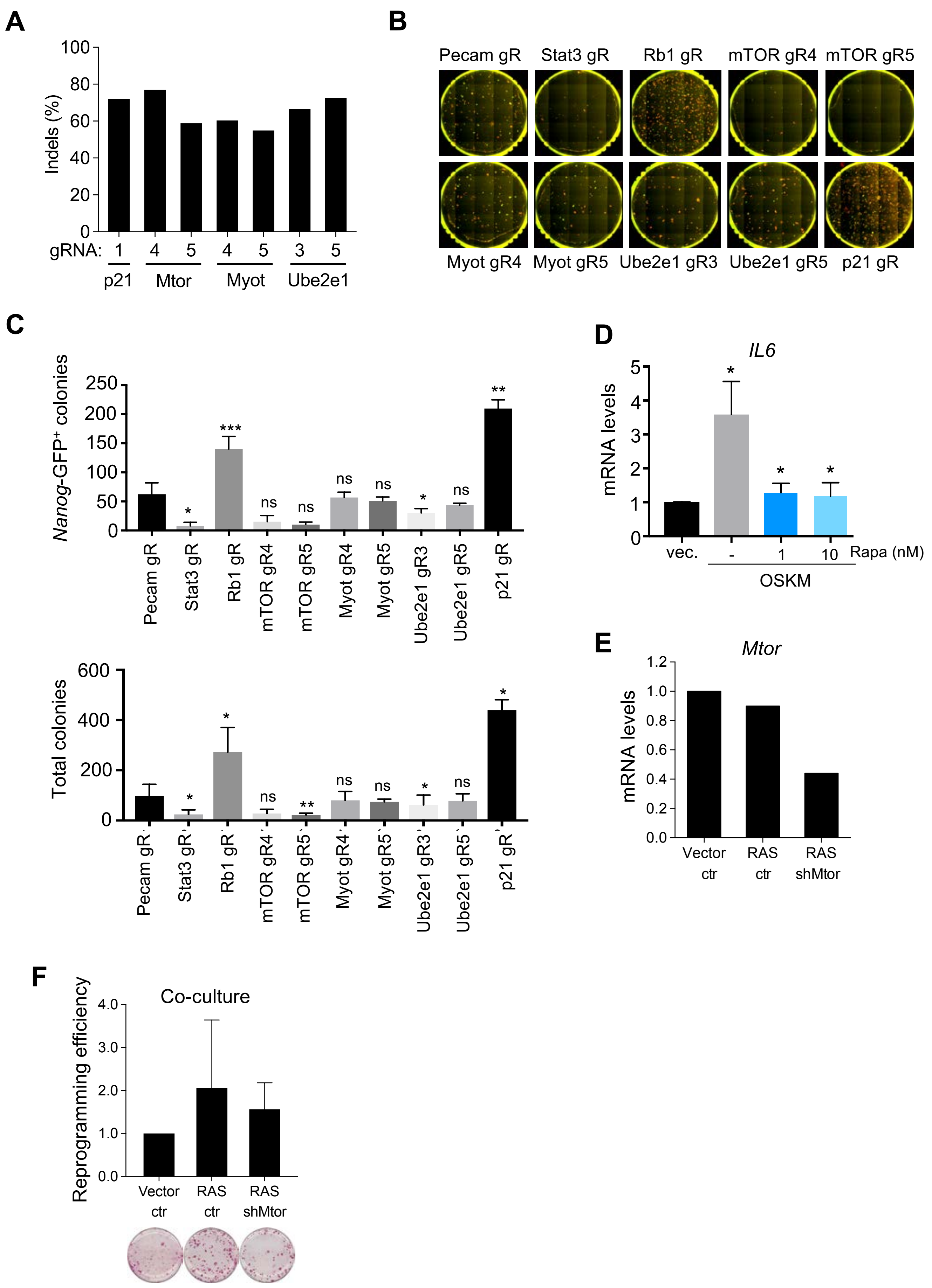
A
Indels (%)
100
80
60
40
20
0
gRNA: 1 4 5 4 5 3 5
p21
Mtor
Myot
Ube2e1
B
Pecam gR
Stat3 gR
Rb1 gR
mTOR gR4
mTOR gR5
Myot gR4
Myot gR5
Ube2e1 gR3
Ube2e1 gR5
p21 gR
C
Nanog-GFP+ colonies
250
200
150
100
50
0
*
***
ns
ns
ns
ns
*
ns
**
Pecam gR
Stat3 gR
Rb1 gR
mTOR gR4
mTOR gR5
Myot gR4
Myot gR5
Ube2e1 gR3
Ube2e1 gR5
p21 gR
Total colonies
600
400
200
0
*
*
ns
**
ns
ns
*
ns
*
D
IL6
mRNA levels
5
4
3
2
1
0
*
*
*
vec.
-
1
10
Rapa (nM)
OSKM
E
Mtor
mRNA levels
1.2
1.0
0.8
0.6
0.4
0.2
0.0
Vector ctr
RAS ctr
RAS shMtor
F
Co-culture
Reprogramming efficiency
4.0
3.0
2.0
1.0
0.0
Vector ctr
RAS ctr
RAS shMtor

SUPPLEMENTAL FIGURE LEGENDS

Supplemental Figure S1. Characterization of the transcriptional program of OSKM-induced senescence.

A-B. GSEA showing enrichment of the indicated signatures in OSKM versus vector (left) and RAS^{G12V} versus vector (right) IMR90 cells. NES, normalized enrichment score; FDR, false discovery rate. **C.** Venn diagram showing common up-regulated genes between Ras vs Vector and OSKM vs Vector. Up-regulated genes were identified using FDR < 0.05 and $\log_2$ FC >1.**D.** GO term analysis of common genes upregulated upon OSKM- and RAS-induced senescence. First, for each senescence type, genes differentially regulated compared to control (Vector) by $\log_2$ FC>1, *P*<0.05, were selected. Next, common genes were uploaded on the online bioinformatics database Metascape (http://metascape.org) for GO term detection and clustering. Same colored dots fall into a similar function category. Titles of selected categories are shown. Only statistically significant categories (*P*<0.05) are shown. **E.** GO term analysis of genes upregulated upon OSKM-induced senescence but not RAS-induced senescence. Genes relevant to OSKM-induced senescence only were used in this instance of GO term clustering, following the criteria used in Figure S1D.

Supplemental Figure S2. Screens for shRNAs blunting OSKM-induced senescence.

A. Timeline of primary genome-wide shRNA enrichment screen and strategy to collect control and experimental samples for subsequent analysis of shRNA

library representation. IMR90 fibroblasts were infected with an OSKM expression vector followed by a pooled human pGIPZ shRNA library in triplicate. At 4 days post-infection, a reference sample was taken (day 0). OSKM- and shRNA-containing cells were selected for 9 days and serially passaged to allow enrichment of shRNAs bypassing OSKM-induced senescence. Genomic DNA was harvested from control and experimental samples; shRNA cassettes were PCR-amplified and analysed for enrichment by deep sequencing. In the primary screen, a gene was classified as a hit if: (1) multiple shRNA constructs targeting the same gene were enriched more than 2-fold, or (2) a single shRNA showed ≥2-fold enrichment in multiple replicates or pools, or (3) a single shRNA showed ≥16-fold enrichment in one replicate. The primary screen resulted in 554 candidate genes. Six shRNAs per candidate gene were cloned for testing in a secondary shRNA screen (3,153 shRNAs in total; Fig. 2). **B**. Proof of principle of enrichment screen. IMR90 fibroblasts were infected with p53 shRNA (shp53) undiluted or diluted at 1/1000 and 1/5000 in non-targeting shRNA vectors. Bypass of the OSKM-induced arrest was assayed by crystal violet staining of cells plated at Day 12 and Day 40 during the screen. **C**. Primary screen data (maximum $\log_2$ fold change in shRNA abundance) in Day 48 versus Day 0 samples of three replicates (black). Highlighted candidates (blue) were selected based on $\log_2$ ratio > 4 in a single replicate or $\log_2$ > 1 in more than 1 replicate. **D**. *TP53*, *CDKN1A*, *MTOR* and *UBE2E1* mRNA levels as determined by qRT-PCR. IMR90 cells were transduced with control or OSKM vector followed by pooled pGIPZ shRNAs as indicated. RNA was extracted at 12 days post-infection to determine knockdown of the corresponding genes. Data was normalized to

OSKM expressing control cells. Error bars represent s.d. of at least 3 independent experiments. ** $p<0.01$; *** $p<0.001$; ns, not significant.

Supplemental Figure S3. Validation of shRNAs identified in the screen.

A. *CDKN1A* (*left*), *MTOR* (*middle*) and *UBE2E1* (*right*) mRNA levels as determined by qRT-PCR. IMR90 cells were transduced with control (grey bars) or OSKM expression vector (black bars) followed by the indicated shRNA vectors or empty control vector (V). Data was normalized to OSKM expressing control cells. Error bars represent s.d. of at least 3 independent experiments. ** $p<0.01$; *** $p<0.001$; ns, not significant. **B**. Quantification of immunofluorescence staining for $p21^{CIP1}$ (*left*), mTOR (*middle*) and UBE2E1 (*right*) in IMR90 cells transduced with control (grey bars) or OSKM expression vector (black bars) followed by the indicated shRNA vectors or empty control vector (V). Error bars represent s.d. of 3 independent experiments for $p21^{CIP1}$ and mTOR and two technical replicates for UBE2E1. * $p<0.05$; ** $p<0.01$; *** $p<0.001$; ns, not significant. **C**. Representative immunofluorescence images of $p21^{CIP1}$ (*top*), mTOR (*middle*) and UBE2E1 (*bottom*) staining in IMR90 cells transduced with OSKM and empty control vector (Vector) or the indicated shRNAs. Nuclei were counterstained with DAPI (bue). Scale bars, 50 µm.

D-E. Representative images of BrdU (**D**) and SA-β-Gal (**E**) IF staining in IMR90 cells transduced with OSKM and empty vector (Vector) or the indicated shRNAs. Nuclei were counterstained with DAPI (bue). Scale bars, 100 µm.

Supplemental Figure S4. Coupling scRNA-Seq to shRNA detection.

A. Mapping of sequencing reads to miRE-shRNA sequence. Only sequencing reads overlapping both the miR30 backbone and shRNA-specific sequence were considered specific for the shRNA. Representative reads mapping to CDKN1A.577 are shown for a given cell. **B**. Correlation between the number of shRNA-specific reads and the number of total reads per sample (results are shown for 300 OSKM/Library cells). **C.** Top marker genes as identified by SC3 are shown for OSKM-expressing cells infected with Vector, shp21, shMTOR, shMYOT and shUBE2E1. Marker genes are highly expressed in one of the clusters and distinguish the cluster from the others. Heat map of the corresponding clusters is shown in Fig. 3G. **D**. Projection of key marker genes *COL14A1*, *NPTX1* and *FST* onto the t-SNE plot shown in Fig. 3H.

Supplemental Figure S5. Using scRNA-Seq to characterize how mTOR regulates OSKM-induced senescence.

A. Violin plots of *MTOR* mRNA expression are shown for single cells expressing MTOR, p21 and other shRNAs versus OSKM and Vector control cells. **B**. Top marker genes as identified by SC3 are shown for each of the clusters shown in Fig. 4C. Cluster 1 is enriched for OSKM-shMTOR cells, cluster 2 for OSKM-expressing cells with p21 or other shRNAs, cluster 3 for Vector control cells (growing) and cluster 4 for OSKM-expressing control cells (senescent). Marker genes are highly expressed in one of the clusters and distinguish the cluster from the others. **C**. Projection of *DLX1* (top), *COL14A1* (middle) and *NPTX1* (bottom) expression onto the t-SNE from Fig. 4D showing differential expression in OSKM-shMTOR, OSKM control (senescent)

and Vector control (growing) sub-populations, respectively. **D**. GSEA showing enrichment of signatures associated with mTOR inhibition, and cell cycle in OSKM-shMTOR versus OSKM control IMR90 cells. NES, normalized enrichment score; FDR, false discovery rate.

Supplemental Figure S6. Induction of CDKIs during OSKM-induced senescence.

A. Heat map showing gene expression of cell cycle genes (Chang et al. 2004) for IMR90 cells infected with vector, RAS, RAS treated with 1nM and 10nM of Rapamycin, OSKM and OSKM treated with 1nM and 10nM of Rapamycin. The gene signature was filtered for genes upregulated in the vector. Both genes and samples were clustered using hierarchical clustering. **B.** Inhibition of mTOR by rapamycin blunts the induction of $p16^{INK4a}$ back to basal levels in OSKM-induced senescence only. IMR90 fibroblasts were infected with empty vector or OSKM- or RAS-expressing vectors and next day treated with DMSO (-) or 1nM rapamycin. At day 10 post-infection, the cells were collected for immunoblot analysis of $p16^{INK4a}$. **C – E.** *CDKN2B*, *CDKN2A* and *CDKN1A* were knocked down with respective siRNAs in OSKM-induced senescent cells. IMR90 fibroblasts were infected with empty vector or OSKM-expressing vector and 2 days later transfected with scramble siRNA (-) or the indicated siRNAs. At day 5 post-infection, the cells were collected for quantitative RT-PCR analysis of target mRNA expression.

Supplemental Figure S7. Effect of mTOR inhibition on reprogramming.

A. Cas9-expressing MEFs were transduced with the indicated lentiviral sgRNA vectors against *Mtor*, *Myot*, *Ube2e1* or empty control vector. Bars represent out-of-frame indel frequencies as measured by TIDE analysis of PCR amplicons spanning the sgRNA target site and using a mock-treated sample as control reference. **B-C.** Reprogramming of Cas9 expressing MEFs was initiated one day after transfection with a *piggyBac* transposon carrying an inducible MKOS cassette and the indicated gRNA expression cassette. Numbers of total and Nanog-GFP$^+$ colonies were counted on day 14. Representative images of the colonies (B) and quantification (C) is shown. This is an expanded version of the data presented in Fig 6A. * $p<0.05$; ** $p<0.01$; *** $p<0.001$; ns, not significant. **D**. Inhibition of mTOR by rapamycin blunted the induction of *IL6* by OSKM. IMR90 fibroblasts were infected with OSKM or Vector and treated with the indicated doses of Rapamycin the next day. After 10 days, RNA was extracted for quantification of *IL6* mRNA levels. Data was normalised to OSKM-infected control cells. Error bars represent s.d. of 3 independent experiments. * $p<0.05$. **E.** *Mtor* mRNA expression levels relative to *Hprt* as determined by qRT-PCR. Wild-type MEFs were transduced with control vector, RAS or RAS and shRNAs against *Mtor*. RNA was extracted at 15 days post-infection. **F.** Reprogramming efficiency of transgenic MKOS MEFs co-cultured with MEFs infected with control vector, RAS, or RAS and shRNAs against *Mtor*. AP+ colonies were counted and data was normalised to vector control cells. Error bars represent s.d. of 3 independent experiments. Images are for a representative experiment.

SUPPLEMENTAL TABLES

Table S1. shRNA and siRNA target sequences

Name	Vector	Target sequence (5'-3')	ID
CDKN1A_a	pGIPZ	ATTCGACTTTGTCACCGAGACA	V3LHS_322231
CDKN1A_b	pGIPZ	ATGGACCTGTCACTGTCTTGTA	V3LHS_322232
CDKN1A_c	pGIPZ	CGACCAGCATGACAGATTTCTA	V3LHS_322234
CDKN1A_d	pGIPZ	CCAGTTTGTGTGTCTTAATTAT	V3LHS_402905
MTOR_m2	pGIPZ	CCAGGCCTATGGTCGAGATTTA	
MTOR_m3	pGIPZ	ATGGGATGTTTTCAGTGGTCAA	
MYOT_a	pGIPZ	CCAGCAAATATTTAGCACTTAA	V3LHS_304529
MYOT_b	pGIPZ	ACTGGATGTCCTTGCAAAAGAA	V3LHS_304528
MYOT_c	pGIPZ	AAAGCTGGAGTGACTACATGTA	V3LHS_304533
MYOT_d	pGIPZ	AAAGAGTTACTTTACTGATAAA	V3LHS_304531
UBE2E1_a	pGIPZ	ACCCCAAGAAGAAGGAGAGTAA	V3LHS_385801
UBE2E1_b	pGIPZ	CTAGCTGAAATGTAGTACAGAA	V3LHS_412316
UBE2E1_c	pGIPZ	ACCCAAGAAGAAGGAGAGTAAA	V3LHS_385802
UBE2E1_d	pGIPZ	CGCTTGTAGTCTGTAAATTTAA	V2LHS_220497
UBE2E1_e	pGIPZ	AGGACAAGAATCTATCATTGTA	V2LHS_171753
shp53	pGIPZ	TCTCTTCCTCTGTGCGCCG	
p21 #1	pRLL	ATCAGTTTGTGTGTCTTAATTA	CDKN1A.577
p21 #2	pRLL	ATCTGGCATTAGAATTATTTAA	CDKN1A.663
p21 #3	pRLL	ATCCCACAATGCTGAATATACA	CDKN1A.1980
MTOR #1	pRLL	ACAGAACAAATACTCAACTAAA	MTOR.8611
MTOR #2	pRLL	CCACCATGTTGTATCAGAATAA	MTOR.8664
MTOR #3	pRLL	CCAGCTAAAGAAGGACATTCAA	MTOR.1710
MYOT #1	pRLL	CCACAAGTAAGAAGTAGATCAA	MYOT.508
MYOT #2	pRLL	CCAGCAAATATTTAGCACTTAA	MYOT.1214
UBE2E1 #1	pRLL	CTAGCTGAAATGTAGTACAGAA	UBE2E1.609
UBE2E1 #2	pRLL	CACAGAAAAGAATGTACATTTA	UBE2E1.620
shp53	pRLL	CGGAGGATTTCATCTCTTGTAT	
CDKN2B_5	siRNA	CTGCTTACTTATGCCATAGAA	SI00288274
CDKN2B_6	siRNA	GAGAGCAATTGTAACGGTTAA	SI00288281
CDKN2A_15	siRNA	TACCGTAAATGTCCATTTATA	SI02664403
CDKN1A_6	siRNA	CAGTTTGTGTGTCTTAATTAT	SI00604898
Mtor #2 (m)	pRLL	CCAGACAGTTGGACTTGTTAAA	Mtor.7848
Mtor #3 (m)	pRLL	ACAGGAGGACATTTGTTCAGAA	Mtor.8297
Mtor #5 (m)	pRLL	CTCCGTTCTATCTCCTTGTCAA	Mtor.5785

m, mouse

Table S2. Antibodies.

Target	Clone	Company	Cat. no.	Application
BrdU	3D4	BD Pharmingen	555627	IF
p21	M-19	Santa Cruz	sc-471	IF
mTOR	7C10	Cell Signaling Technology	2983	IF
UBE2E1		Abcam	ab36980	IF
p16	JC8	CRUK	n/a	IF, WB

IF, immunofluorescence

Table S3. Primers used for RT-qPCR.

Name	Forward (5'-3')	Reverse (5'-3')
CDKN2B	GAATGCGCGAGGAGAACAAG	CCATCATCATGACCTGGATCG
CDKN2A	CGGTCGGAGGCCGATCCAG	GCGCCGTGGAGCAGCAGCAGCT
CDKN1A	CCTGTCACTGTCTTGTACCCT	GCGTTTGGAGTGGTAGAAATCT
p53	CCGCAGTCAGATCCTAGCG	AATCATCCATTGCTTGGGACG
MTOR	TCGCTGAAGTCACACAGACC	CTTTGGCATATGCTCGGCAC
UBE2E1	GGAGTCCAGCACTAACCATTTCT	GGCAATACTTCCCACCAAGGG
IL6	CCAGGAGCCCAGCTATGAAC	CCCAGGGAGAAGGCAACTG
GAPDH	GGAGCGAGATCCCTCCAAAAT	GGCTGTTGTCATACTTCTCATGG
mMtor	CCAATGAGAGGAAGGGTGGCATC	GGACGCCATTTCCATGACAACTG
mHprt	CACAGGACTAGAACACCTGC	GCTGGTGAAAAGGACCTCT

m, mouse

Table S4. sgRNA target and TIDE PCR primer sequences.

Name	sgRNA Target (5'-3')	TIDE PCR primers (5'-3')
Mtor #4	GCGGGGTAGAACTCGTCCAG	*F*: GAACTGTGACGCTGAGAAGCTA<br>*R*: TCCAGTGGGATGGAGTAGAACT<br>*Seq*: GAGTACTTACTTCCCGGATGGC
Mtor #5	GTCTGATTCTCACCACGCAG	*F*: TGGCTTTGTCAGTCACAACTTT<br>*R*: TTATAGGGGTGTCCCACCATAG<br>*Seq*: CAAGGATGACAGGGGTGTGT
Myot #4	GAAAACATGTCGATCGAAGA	*F*: GAGCTACCTCAAAGGGGATTTT<br>*R*: AATGGACAACTACAGATGCGTG<br>*Seq*: AGGCTGTGGACTTTGTAGCA
Myot #5	GCGAGCCATCTTCTCCTCGT	*F*: AATTCCAACTAAGCCTGTGCTC<br>*R*: ACAGCTCTTGAGTTTGCCTTTC<br>*Seq*: TAGCAAGCTCAGAAACAGGGA
Ube2e1 #3	GCTTACCTGCAGTTTGGCGG	*F*: TTCATTCACGGGTGAGATACTG<br>*R*: AAGAATCTGACGGTGTTGGTCT<br>*Seq*: TGCCCACTCCATGAAAATCC
Ube2e1 #5	GGGAGACTCCTTACCTTTGG	*F*: AATGGGTCTGGTGTTCTTCTGT<br>*R*: GTAGCTGCAAATTTTTGATCCC<br>*Seq*: AACCCAACCAACAGTCATCACA
p21	GATTGCGATGCGCTCATGGC	
Pecam1	GAGAACTCTAACTTCGGCTT	
Stat3	GGATGACTAAGGGCCGGTCC	
Rb1	GCATCACACGGTAATACAAT	

F, Forward; *R*, Reverse; *Seq*, Sequencing

SUPPLEMENTAL MATERIALS AND METHODS

Retroviral and lentiviral infection

For retroviral transduction, HEK293T cells were transfected with retroviral MSCV-neo vectors expressing a polycistronic cassette encoding Oct4, Sox2, Klf4, and c-Myc (OSKM)(Carey et al. 2009) or constitutively active RAS (H-RAS^{G12V}), and packaging vectors using 1 mg/ml linear polyethylenimine transfection reagent (PEI 25000; Polysciences). Viral supernatants were collected in three rounds starting 48 h after transfection, filtered and added to IMR90 fibroblasts plated the day before at a density of 10^6 cells per 10 cm dish or 3.5 x 10^5 cells per 6 cm dish in the presence of 5 µg/ml polybrene. After 24 h of retroviral infection, media was either replaced with fresh media containing rapamycin (when indicated) or with 1:4 diluted lentiviral supernatant generated from shRNA vectors for 4 h before replacing the media. Three days later, cells were passaged and cultured for 9 days in the presence of 0.75 µg/ml puromycin (InvivoGen) or 400 µg/ml neomycin (Geneticin, G418; Gibco) to select for infected cells. After selection, cells were plated for growth assays, SA-β-gal and BrdU incorporation assays or lysed in Trizol for RNA extraction.

shRNA libraries and screening

For the initial screen, we used a pGIPZ human genome-wide shRNA library consisting of ~58,000 lentiviral constructs. The library was divided into 12 pools, packaged into lentiviruses and introduced into OSKM-infected IMR90 fibroblasts in triplicate at a multiplicity of ~0.3. Samples from screening pools

(10^6 cells in triplicate) were collected three days post-infection (reference, day 0) and then at regular intervals over a 48-day culture period.

Based on the results of the initial screen, a second library consisting of 3,153 miRE-based shRNAs targeting 554 candidate genes (average coverage of six shRNAs per gene) was constructed using sensor-based shRNA predictions (Fellmann et al. 2011; Fellmann et al. 2013). 136-mer oligonucleotides (each containing a 97 nt miRE-shRNA fragment, an EcoRI cloning site and a 20 nt adaptor site) were synthesized on an oligonucleotide array (MYcroarray) and pooled for cloning (Cleary et al. 2004; Fellmann et al. 2011). The pool of oligonucleotides was PCR-amplified and cloned through XhoI/EcoRI sites into the pRLL-SFFV-GFP-miRE-PGK-Puro vector (Fellmann et al. 2013). The secondary library was introduced into OSKM-infected IMR90 fibroblasts in duplicate at a multiplicity of ~0.3. Samples were collected three days post-infection (reference, day 0) and then at regular intervals over a 37-day culture period. The screen was repeated twice. On day 56 during the second repeat screen, OSKM-infected library cells from one replicate were sorted into three 96-well plates by flow cytometry (FACS Aria, BD) for single cell RNA-seq analysis using the Smart-seq2 protocol (Picelli et al. 2014).

BrdU incorporation assays

For BrdU immunofluorescence, cells (2-3x10^3) were plated in 96-well plates in duplicate and cultured for 5 days before 50 μM BrdU (5-Bromo-2′-deoxyuridine) was added for 18-20 h. Cells were fixed, permeabilised and incubated with mouse anti-BrdU antibody (1:2000; BD Pharmingen, 555627)

in the presence of DNase I (0.5 U/µl; Sigma, D4527) and 1 mM $MgCl_2$ in BS for 30 min at room temperature.

Crystal violet staining

Cells were plated at low density (2 x 10^5 cells per 10 cm dish or 7 x 10^4 cells per 6 cm dish) and cultured for 14 days. Cells were fixed in 0.5% glutaraldehyde solution in PBS for 30 min and stained with 0.2% crystal violet solution in H_2O for at least 30 min.

SA-β-galactosidase staining

For fluorescence-based detection of SA-β-gal activity, cells (8x10^3) were plated in 96-well plates in triplicate. The next day, fresh media was added with 100 nM bafilomycin A1 (Sigma, B1793) and 100 µM DDAO galactoside (9H-(1,3-Dichloro-9,9-Dimethylacridin-2-One-7-yl) β-D-Galactopyranoside; Molecular Probes, D6488) for 2 h at 37˚C, 5% CO_2. Cells were washed with PBS, fixed in 4% formaldehyde for 15 min and nuclei were stained with DAPI before image acquisition. For cytochemical detection of SA-β-gal activity, cells were plated at 2.5-3x10^5 per 6 cm dish. The next day, cells were fixed in 0.5% glutaraldehyde solution in PBS for 15 min and stained for 16 h at room temperature as described previously (Debacq-Chainiaux et al. 2009).

High content analysis

Image acquisition was performed using an automated high throughput microscope (IN Cell Analyzer 2000, GE Healthcare) with 10x or 20x objectives. Image processing was performed using the IN Cell Investigator software

(v3.7; GE Healthcare). DAPI staining of the nuclei was used to identify nuclear area and number of cells. The nuclei were segmented using top-hat segmentation, specifying a minimum nuclear area of 100 μm^2. To define the cell area, a collar segmentation approach was used with a border of 3 μm around DAPI staining or alternatively, multiscale top-hat segmentation was used to detect cytoplasmic SA-β-gal staining intensity. Each cell was assigned a nuclear and cell intensity value depending on the protein being studied. Intensities of all cells in a sample were plotted in a histogram to set a threshold filter and determine positive and negative populations.

Cell isolation by limited dilution

Cells were stained with Hoechst 33342 and Propidium Iodide (Thermo Fisher) for 20 minutes. The cell viability and density was checked using a Moxi Mini cell counter (ORFLO). Cells were diluted to achieve a density of 1 cell per 50 nl in a final dispensing mix which contained a diluent, RNAsin (New England Biolab) and 0.35X PBS (without Ca^{++} and Mg^{++}, pH 7.4, Thermo Fisher). A 384-well source plate with 8 designated wells containing cell suspensions, positive and negative controls, and fiducial mix (fluorescent dye permitting image alignment confirmation) was placed in the ICELL8™ MultiSample NanoDispenser (MSND) (WaferGen). Each of 8 sample source wells in the 384-source plate was sampled by 1 of the 8 dispensing tips. Cells, positive controls, negative controls, and fiducial mix were dispensed onto one chip within 16 minutes. Total RNA (~10 pg) from IMR90 cells was dispensed into selected nanowells and used as in-process positive controls.

Microchip imaging and selection of single-cell-containing nanowells

After dispensing, each chip was sealed and centrifuged at 300 g for 5 minutes at 4°C before imaging with the ICELL8™ Imaging Station (WaferGen). A total of 288 images, 144 each for Hoechst 33342 and for Propidium Iodide were captured. Each image comprised the picture of 36 wells. Following imaging (~7 minutes), the microchip was stored at -80°C for at least 45 minutes or until ready for further processing.

Microchip images were analyzed using CellSelect™ software (WaferGen) to determine the viability and number of cells present in each nanowell. Using the default configuration, CellSelect identified nanowells that have one cell in channel 1 (Hoechst) and no cells in channel 2 (PI). Nanowells that had one bright cell and additional dim cells or debris were further excluded. Nanowells that contained only one cell were selected as candidates and additional visual inspection was performed to confirm the presence of single viable cells.

Single-cell cDNA generation for the ICELL8™ experiments

IMR90 fibroblasts were retrovirally infected with OSKM or control vector, followed by lentiviral infection with the indicated shRNA constructs, and then cultured in selection media as described earlier. Single cells were dispensed on an ICELL8™ microchip with pre-printed barcoded oligonucleotides (WaferGen Biosystems). Live, single cells were selected based on positive Hoechst 33342 and negative propidium iodide staining using the CellSelect™ software (WaferGen Biosystems). Chips were then centrifuged at 3,800 g for 5 minutes at 4°C and transferred to a thermocycler with a program of 72°C for 3 minutes and 4°C forever to anneal pre-printed oligonucleotides to polyA

mRNAs. The microchips were centrifuged as previously before placing them into the MSND. RT-PCR reagents (Takara Bio) contained the following components: dNTP mix, Triton-X-100, $MgCl_2$, dithiothrietol (DTT), betaine, SeqAmp™ PCR buffer, SMARTScribe™ First Strand buffer, SMARTScribe™ reverse transcriptase and SeqAmp™ DNA polymerase. The RT-PCR mastermix was dispensed at 1x concentration in each selected well of the ICELL8 chip and was supplemented with 0.8 μM Template Switch Oligo (TSO) and 0.2 μM amplification primer (AP) final concentration. The microchips were spun down and transferred to a thermocycler with a program of 42°C for 90 minutes, 2 cycles at 50°C and 42°C for 2 minutes each to perform cDNA synthesis and a heat-kill step for RT at 70°C for 15 minutes followed by a PCR program of 95°C for 1 min, 18-24 cycles of 98°C for 10 seconds, 65°C for 30 seconds, 68°C for 3 minutes, and 1 cycle of 72°C for 10 minutes and 4°C forever. The number of cycles of amplification depends on cell size and the amount of total RNA in each cell with smaller cells requiring more cycles of amplification. Post reaction chips were inverted and centrifuged (3,800 g 10 minutes at 4°C) to simultaneously collect and pool well contents into a single microcentrifuge collection tube. Double-stranded cDNA was cleaned by the DNA Clean & Concentrator^{TM}-5 kit (Zymo Research). Amplicons were purified using Agencourt AMPure XP magnetic beads (Beckman Coulter). Library quality was assessed using a Bioanalyzer High Sensitivity DNA chip (Agilent Technologies) and quantity was determined by a Qubit High Sensitivity kit (Thermo Fisher Scientific).

RNA-Seq library construction and sequencing for the ICELL8™ experiments

1ng of cDNA was used for library construction using the Nextera XT kit (Illumina) per manufacturer's instruction. A custom-made Nextera P5 (WaferGen) and a P7 index primer provided by the Nextera XT kit (Illumina) were used to amplify the "tagmented" fragments. Libraries were purified and size selected using Agencourt AMPure XP magnetic beads (Beckman Coulter) to obtain an average library size of 500 bp. Libraries were sequenced asymmetrically (26 bp for read 1, 166 bp for read 2) on a HiSeq 2500 (Illumina) in rapid run mode. Initial demultiplexing was performed using CASAVA v1.8 allowing 0 mismatches, which generated 208.68 million reads passing filter for 460 samples (OSKM/Lib cells and controls; Fig. 3A-D) or 133.94 million reads passing filter for 310 samples (OSKM/shRNA cells and controls; Fig. 3E-H).

Preparation of Smart-Seq2 libraries for scRNA-Seq

Smart-seq2 libraries were prepared according to the previously described protocol (Picelli et al. 2014) with a few modifications. At step 5, 0.1 µl of ERCC RNA Spike-in mix (10^{-5} diluted; Life Technologies, 4456740) was added with 0.1 µl of 100 µM oligo-dT primer, 1 µl of dNTP mix and 0.8 µl of H_2O, yielding the same concentrations of primer and oligo as originally reported. We used 18 cycles for the pre-amplification PCR in step 14. Starting at step 28 in the Smart-seq2 protocol, we performed the Nextera XT reactions in 4 x smaller volumes using 2 µl of undiluted cDNA. In step 33, 12 cycles were used for the final enrichment PCR. After the enrichment, PCR in step 33

for 12 cycles, 2 µl of each 96-well was pooled to form a single library, which was then purified using AMPure XP beads. The resulting library was quantified by Qubit dsDNA HS Assay (Life Technologies) and Bioanalyzer (Agilent Technologies) readings. Pooled libraries were then subjected to 100bp paired-end sequencing per standard protocols for the Illumina HiSeq 2500. Initial demultiplexing was performed using CASAVA v1.8 allowing 0 mismatches, which generated 305.38 million reads for 384 samples.

scRNA-Seq data analysis

FASTQ files were generated from Illumina base call files using bcl2fastq2 conversion software (v2.17). Sequence reads were aligned to the Ensembl GRCh37 genome build and gene models retrieved from Illumina's iGenomes using TopHat2 (v2.0.11).

For transcriptome analysis, aligned reads were counted within exons using Rsubread (v1.22.3) using default parameters without strand specificity. Differential gene expression analysis of single cell data was performed using the scde (v2.0.1) and DESeq2 (v1.12.4) packages, with data quality and default filtering performed using the Scatter package (v1.1.8). Normalized counts from DESeq2 were used for single cell clustering, PCA and tSNE analysis. For shRNA assignment, reads were aligned to shRNA specific sequences using BWA with no multiple mapping. shRNAs were assigned to a cell when more reads than an arbitrarily designed cut-off were found within a unique shRNA. t-SNE analysis was performed using the Rtsne package (v0.11) and overlaid normalized counts were plotted using the ggplot2 package (v2.2.0). Differential expression analysis of bulk cell data was

performed using DESeq2 (v1.12.4) package with no additional filtering applied. Functional enrichment analysis was performed using GSEA (v2.2.1) with pre-ranked lists from MSigDB (v4).

For unsupervised clustering of scRNA-Seq data, counts of uniquely mapped reads in every protein coding gene were calculated using SeqMonk (www.bioinformatics.bbsrc.ac.uk/projects/seqmonk) and exported for downstream analysis. Cells were filtered based on a minimum number of 2000 expressed genes per cell. Clusters and marker genes were obtained using the SC3 package (Kiselev et al. 2017).

Reprogramming experiments with TNG MKOS MEFs

TNG MKOS MEFs were seeded onto 6-well plates coated with 0.1% gelatin (Sigma, G1393) in PBS at a density of $2x10^4$ MEFs per 6 well in MEF medium. The following day, the media was replaced by reprogramming medium (MEF medium supplemented with 1 µg/ml doxycycline (Sigma, D9891), 10 µg/ml Vitamin C (L-ascorbic acid; Sigma, A4403) and 1,000 U/mL ESGRO Leukemia Inhibitory Factor (LIF; Millipore ESG1107). Medium was replenished every 2 days. After 14 days, colonies were stained using an Alkaline Phosphatase (AP) detection kit (Millipore, SCR004). Cells were treated with rapamycin at the indicated doses for the first three or six days. For the reprogramming experiments with gene knockout, *Rosa26-Cas9* knock-in mice were crossed with *Nanog*-GFP reporter mice (Chambers et al. 2007). Cas9 expressing *Nanog*-GFP MEFs from E12.5 embryos were reprogrammed with *piggyBac* transposon carrying tetO-*MKOS-ires-mOrange* cassette (Kaji et al. 2009) as well as U6-gRNA expression cassette, by co-

transfection with pCMV-hyPBase (Yusa et al. 2011) and PB-CA-rtTA Adv (Woltjen et al. 2009). The MEFs were plated at $1.5x10^5$ cells per well in a 6-well plate, and 24 hours later transfected with FugeneHD (Promega) as per manufacturer's instructions. One day after transfection, reprogramming was initiated with ES media containing 1 µg/ml Dox (Clontech), 10 µg/ml Vitamin C (Sigma) and 100 U/mL human LIF, in the presence or absence of 500 nM Alk5i (A83-01, Tocris) and/or 5 nM Rapamycin (Sigma). Whole well images were taken with Celigo S imaging cytometer (Nexcelome). Sequences of gRNAs are shown in Table S4.

TIDE analysis

Cas9-expressing MEFs were transduced with U6-gRNA-PGKpuro-2A-BFP lentiviral vectors (sequences are listed in Table S4). After 6 days, the number of BFP-positive cells was assessed by flow cytometry (>90%) and genomic DNA (~2 x 105 cells) was extracted using the Quick-gDNA MicroPrep kit (Zymo Research). PCR reactions were carried out with 50 ng genomic DNA in MyTaq Red mix (Bioline) as described in Brinkman et al. (2014 Brinkman NAR). PCR products were purified using the QIAquick PCR Purification Kit (Qiagen) and prepared for sequencing using the primers listed in Table S4. Genome editing efficiency (excluding in frame indels) was determined by comparing the sequence traces from control and sgRNA infected cells using the TIDE web tool.

siRNA experiments

siRNAs were purchased from Qiagen lyophilised in a Flexitube®. Targeting

sequences are provided in Table S1. For immunofluorescence analysis, 2 days after retroviral transduction of empty, OSKM- or RAS-expressing vector, IMR90 cells in suspension (100 µl) were reverse transfected with siRNAs on a well of a 96-well plate. The suspension media was DMEM supplemented with 10% FBS only. The transfection mix for each sample well contained 0.1 µL DharmaFECT™ 1 (GE Healthcare) in 17.5 µL plain DMEM mixed with 3.6 µL siRNA 30 min prior to cell seeding. 18 hours after transfection, allowing target cells to adhere, the media were replaced with fresh complete media, containing neomycin (G418; 400 µg/mL) for selection of cells carrying the transgene. The cells were fixed at the specified time-point with 4% PFA (w/v). For mRNA analysis, the procedure was identical but scaled up 20 times to fit a 6-well plate. The cells were harvested by scraping in 0.8 ml TRIzol® RNA isolation reagent (Ambion) per well.

SUPPLEMENTAL REFERENCES

Brinkman EK, Chen T, Amendola M, van Steensel B. 2014. Easy quantitative assessment of genome editing by sequence trace decomposition. *Nucleic Acids Res* **42**: e168.

Carey BW, Markoulaki S, Hanna J, Saha K, Gao Q, Mitalipova M, Jaenisch R. 2009. Reprogramming of murine and human somatic cells using a single polycistronic vector. *Proc Natl Acad Sci U S A* **106**: 157-162.

Chambers I, Silva J, Colby D, Nichols J, Nijmeijer B, Robertson M, Vrana J, Jones K, Grotewold L, Smith A. 2007. Nanog safeguards pluripotency and mediates germline development. *Nature* **450**: 1230-1234.

Cleary MA, Kilian K, Wang Y, Bradshaw J, Cavet G, Ge W, Kulkarni A, Paddison PJ, Chang K, Sheth N et al. 2004. Production of complex nucleic acid libraries using highly parallel in situ oligonucleotide synthesis. *Nat Methods* **1**: 241-248.

Debacq-Chainiaux F, Erusalimsky JD, Campisi J, Toussaint O. 2009. Protocols to detect senescence-associated beta-galactosidase (SA-betagal) activity, a biomarker of senescent cells in culture and in vivo. *Nat Protoc* **4**: 1798-1806.

Fellmann C, Zuber J, McJunkin K, Chang K, Malone CD, Dickins RA, Xu Q, Hengartner MO, Elledge SJ, Hannon GJ et al. 2011. Functional identification of optimized RNAi triggers using a massively parallel sensor assay. *Mol Cell* **41**: 733-746.

Kaji K, Norrby K, Paca A, Mileikovsky M, Mohseni P, Woltjen K. 2009. Virus-free induction of pluripotency and subsequent excision of reprogramming factors. *Nature* **458**: 771-775.

Kiselev VY, Kirschner K, Schaub MT, Andrews T, Yiu A, Chandra T, Natarajan KN, Reik W, Barahona M, Green AR et al. 2017. SC3: consensus clustering of single-cell RNA-seq data. *Nat Methods* **14**: 483-486.

Woltjen K, Michael IP, Mohseni P, Desai R, Mileikovsky M, Hamalainen R, Cowling R, Wang W, Liu P, Gertsenstein M et al. 2009. piggyBac transposition reprograms fibroblasts to induced pluripotent stem cells. *Nature* **458**: 766-770.

Yusa K, Zhou L, Li MA, Bradley A, Craig NL. 2011. A hyperactive piggyBac transposase for mammalian applications. *Proc Natl Acad Sci U S A* **108**: 1531-1536.
